# Machine learning derivation of four computable 24-h pediatric sepsis phenotypes to facilitate enrollment in early personalized anti-inflammatory clinical trials

**DOI:** 10.1186/s13054-022-03977-3

**Published:** 2022-05-07

**Authors:** Yidi Qin, Kate F. Kernan, Zhenjiang Fan, Hyun-Jung Park, Soyeon Kim, Scott W. Canna, John A. Kellum, Robert A. Berg, David Wessel, Murray M. Pollack, Kathleen Meert, Mark Hall, Christopher Newth, John C. Lin, Allan Doctor, Tom Shanley, Tim Cornell, Rick E. Harrison, Athena F. Zuppa, Russell Banks, Ron W. Reeder, Richard Holubkov, Daniel A. Notterman, J. Michael Dean, Joseph A. Carcillo

**Affiliations:** 1 Graduate School of Public Health, University of Pittsburgh, Pittsburgh, PA USA; 2 Division of Pediatric Critical Care Medicine, Department of Critical Care Medicine, Children’s Hospital of Pittsburgh, Center for Critical Care Nephrology and Clinical Research Investigation and Systems Modeling of Acute Illness Center, Faculty Pavilion, UPMC Children’s Hospital of Pittsburgh, University of Pittsburgh, Suite 2000, 4400 Penn Avenue, Pittsburgh, PA 15421 USA; 3 Department of Computer Sciences, University of Pittsburgh, Pittsburgh, PA USA; 4 Department of Pediatrics, University of Pittsburgh, Pittsburgh, PA USA; 5 Department of Anesthesiology, Children’s Hospital of Philadelphia, Philadelphia, PA USA; 6 Division of Critical Care Medicine, Department of Pediatrics, Children’s National Hospital, Washington, DC USA; 7 Division of Critical Care Medicine, Department of Pediatrics, Children’s Hospital of Michigan, Detroit, MI USA; 8 Central Michigan University, Mt. Pleasant, MI USA; 9 Division of Critical Care Medicine, Department of Pediatrics, The Research Institute at Nationwide Children’s Hospital Immune Surveillance Laboratory, and Nationwide Children’s Hospital, Columbus, OH USA; 10 Division of Critical Care Medicine, Department of Anesthesiology and Critical Care Medicine, Children’s Hospital Los Angeles, Los Angeles, CA USA; 11 Division of Critical Care Medicine, Department of Pediatrics, St. Louis Children’s Hospital, St. Louis, MO USA; 12 Division of Critical Care Medicine, Department of Pediatrics, C. S. Mott Children’s Hospital, Ann Arbor, MI USA; 13 Division of Critical Care Medicine, Department of Pediatrics, Mattel Children’s Hospital at University of California Los Angeles, Los Angeles, CA USA; 14 University of Utah, Salt Lake City, UT USA; 15 Princeton University, Princeton, NJ USA

**Keywords:** Severe sepsis, Multiple organ failure, Immunoparalysis-associated multiple organ failure, Thrombocytopenia-associated multiple organ failure, Macrophage activation syndrome, Sequential multiple organ failure, Hyperferritinemic sepsis

## Abstract

**Background:**

Thrombotic microangiopathy-induced *thrombocytopenia-associated multiple organ failure* and hyperinflammatory *macrophage activation syndrome* are important causes of late pediatric sepsis mortality that are often missed or have delayed diagnosis. The National Institutes of General Medical Science sepsis research working group recommendations call for application of new research approaches in extant clinical data sets to improve efficiency of early trials of new sepsis therapies. Our objective is to apply machine learning approaches to derive computable 24-h sepsis phenotypes to facilitate personalized enrollment in early anti-inflammatory trials targeting these conditions.

**Methods:**

We applied consensus, *k*-means clustering analysis to our extant PHENOtyping sepsis-induced Multiple organ failure Study (PHENOMS) dataset of 404 children. 24-hour computable phenotypes are derived using 25 available bedside variables including C-reactive protein and ferritin.

**Results:**

Four computable phenotypes (PedSep-A, B, C, and D) are derived. Compared to all other phenotypes, PedSep-A patients (*n* = 135; 2% mortality) were younger and previously healthy, with the lowest C-reactive protein and ferritin levels, the highest lymphocyte and platelet counts, highest heart rate, and lowest creatinine (*p* < 0.05); PedSep-B patients (*n* = 102; 12% mortality) were most likely to be intubated and had the lowest Glasgow Coma Scale Score (*p* < 0.05); PedSep-C patients (*n* = 110; mortality 10%) had the highest temperature and Glasgow Coma Scale Score, least pulmonary failure, and lowest lymphocyte counts (*p* < 0.05); and PedSep-D patients (*n* = 56, 34% mortality) had the highest creatinine and number of organ failures, including renal, hepatic, and hematologic organ failure, with the lowest platelet counts (*p* < 0.05). PedSep-D had the highest likelihood of developing *thrombocytopenia-associated multiple organ failure* (Adj OR 47.51 95% CI [18.83–136.83], *p* < 0.0001) and *macrophage activation syndrome* (Adj OR 38.63 95% CI [13.26–137.75], *p* < 0.0001).

**Conclusions:**

Four computable phenotypes are derived, with PedSep-D being optimal for enrollment in early personalized anti-inflammatory trials targeting thrombocytopenia-associated multiple organ failure and macrophage activation syndrome in pediatric sepsis. A computer tool for identification of individual patient membership (www.pedsepsis.pitt.edu) is provided. Reproducibility will be assessed at completion of two ongoing pediatric sepsis studies.

**Supplementary Information:**

The online version contains supplementary material available at 10.1186/s13054-022-03977-3.

## Introduction

Severe sepsis defined by infection and organ failure contributes to 1 of 5 deaths globally, with 3 million per year occurring in children [1]. While there is evidence that sepsis mortality increases if treatment is delayed [2, 3], several studies in high-income countries where rapid access to intensive care support has been provided, have demonstrated patterns of mortality even in previously healthy children with timely treatment [4–6]. This indicates that dysregulated host immune activation could be targetable in the pediatric intensive care unit (PICU) [7–19]. Among such conditions are immune depression leading to immunoparalysis-associated MOF (IPMOF) [7, 8, 14, 15], thrombotic microangiopathy leading to thrombocytopenia-associated MOF (TAMOF) [9, 10, 14, 15], and hyperinflammatory macrophage activation syndrome (MAS) driven either by uncontrolled lymphoproliferation manifest as sequential liver failure-associated MOF (SMOF) [11, 14, 15] or by macrophage activation without lymphoproliferation manifest as combined hepatobiliary dysfunction and disseminated intravascular coagulation [12–15]. In the PHENOtyping pediatric sepsis-induced Multiple organ failure Study (PHENOMS) [15], we previously reported that these conditions developed at a median of day 3 to 7 of sepsis, with TAMOF and MAS demonstrating 46% mortality, and IPMOF 16% mortality [15]. Anti-inflammatory therapies used to reverse TAMOF and MAS include methylprednisolone, intravenous immunoglobulin (IVIG) and plasma exchange [9, 16–19]. Our clinical trials challenge is to identify these at-risk children for early enrollment when personalized therapies have their greatest likelihood to succeed.

The NIGMS (https://loop.nigms.nih.gov/2019/05/recommendations) sepsis research working group recommendations call for use of new clinical research approaches in extant clinical data sets to characterize septic patients and improve the efficiency of early trials of new sepsis treatments. In this manuscript, we test the hypothesis that machine learning methods previously used in adults [20] could be applied to available bedside clinical variables including C-reactive protein and ferritin in the extant PHENOMS dataset [15] to derive 24-h computable sepsis phenotypes [20–22] that identify children at risk for development of TAMOF and MAS for enrollment in early personalized anti-thrombotic and anti-inflammatory clinical trials.

## Materials and methods overview

We analyzed blood samples and clinical data obtained from our previously published PHENOMS study [15]. Approval was obtained from The University of Utah Institutional Review Board, Central IRB # 70976. Written informed consent was obtained from one or more parents/guardians for each child. Assent was garnered when the child was able. Patients were enrolled from 2015–2017. The CONSORT diagram (Additional file 1: Fig. S1) and details of the parent clinical study protocol have been previously published [15]. Three consented and enrolled children who were excluded from reporting in the parent study manuscript because there was a cap of 81 patients to maximize equalization in enrollment among the centers are additionally included in this machine learning manuscript. Children qualified for enrollment in PHENOMS if they (1) were between the ages of 44 weeks gestation to 18 years of age; (2) were suspected of having infection meeting two or more of four systemic inflammatory response criteria [23]; (3) had one or more organ failures [24]; and (4) had an indwelling arterial or central venous catheter [15]. Children were excluded from enrollment if there was not a commitment to aggressive PICU care. Clinical data and blood samples measuring C-reactive protein, Ferritin, sFASL, ADAMTS 13 activity, and whole blood ex vivo TNF response to endotoxin were obtained on day one and twice weekly until 28 days in the PICU in the parent study [15].

**Fig. 1:**
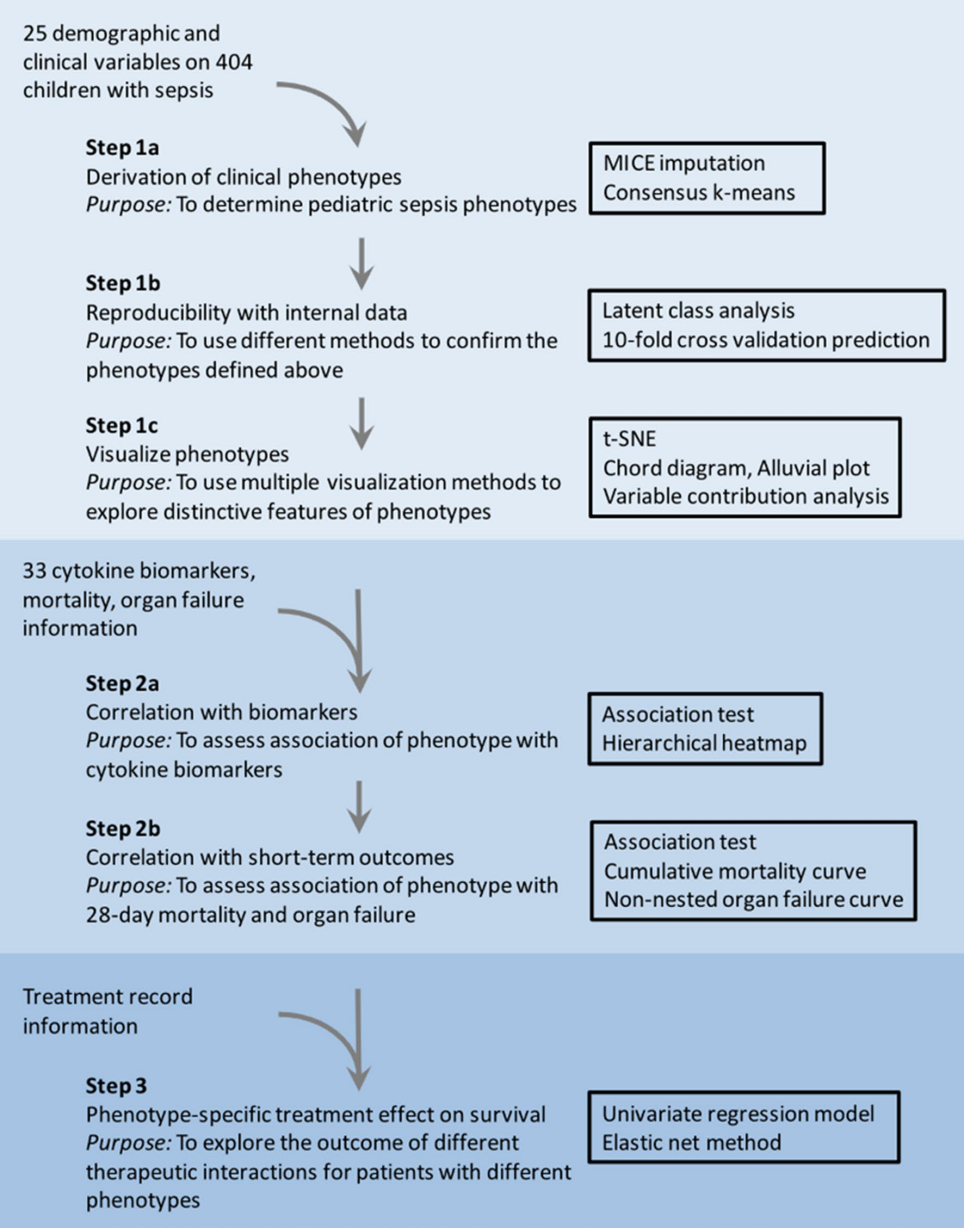
Overview of machine learning, visualization, and statistical methods applied to the PHENOMS pediatric sepsis data set

The parent study was not designed with a plan for performing post hoc machine learning analysis. To minimize inherent selection bias, we set the a priori elements and derived findings before performing the machine learning analysis. The a priori elements included all data available and previously published in the parent study [15], all patients enrolled in the CONSORT diagram (Additional file 1: Fig. S1), and additional measurements of multiple cytokines. The derived machine learning approach methods and findings are illustrated in Fig. 1 and were designed and set a priori to address the following recommendations of DeMerle and colleagues [25].

**Fig. 2:**
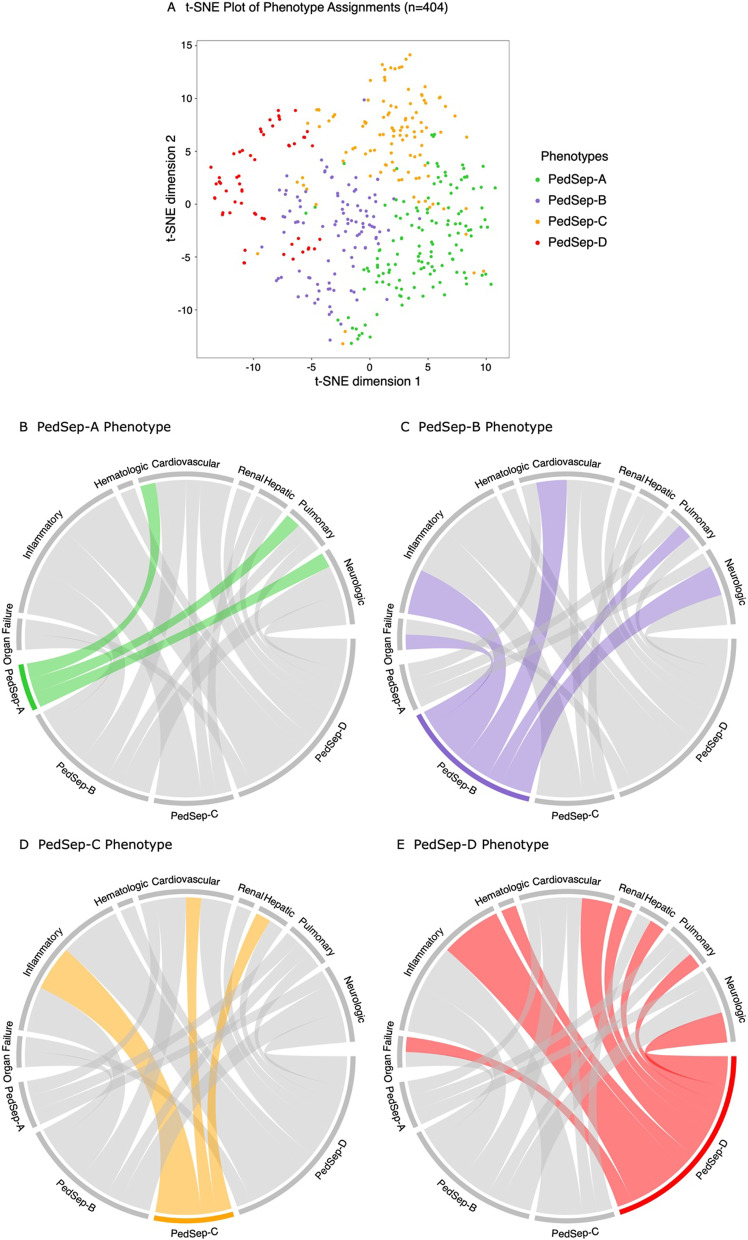
24-hour phenotype distribution and chord plot. In panel **A**, visualization of phenotypes using t-distributed stochastic neighbor embedding (t-SNE) technique with phenotypes shown in color from the consensus *k*-means clustering analysis visualizes distinction among four phenotypes. In panels **B**–**E**, each phenotype is highlighted separately and the ribbons connect to the different patterns of clinical variables and organ system dysfunctions on the top of the circle (inflammation = low temperature, high temperature, max CRP, max ferritin; organ failure = total OFI; pulmonary = pulmonary OFI, intubation; cardiovascular = high heart rate, low systolic blood pressure, cardiovascular OFI; renal = high creatinine, renal OFI; hepatic = hepatic OFI; hematologic = low hemoglobin, low platelets, hematologic OFI; neurologic = Low Glasgow Coma Score Scale, central nervous system OFI). The chords connect from an individual phenotype to a category if the group mean involvement of the variables differs from the overall mean for the entire cohort (see Table 1) specifically lower for low temperature, systolic blood pressure, hemoglobin, platelets, and Glasgow Coma Scale Score, but higher for all other variables

**Fig. 3:**
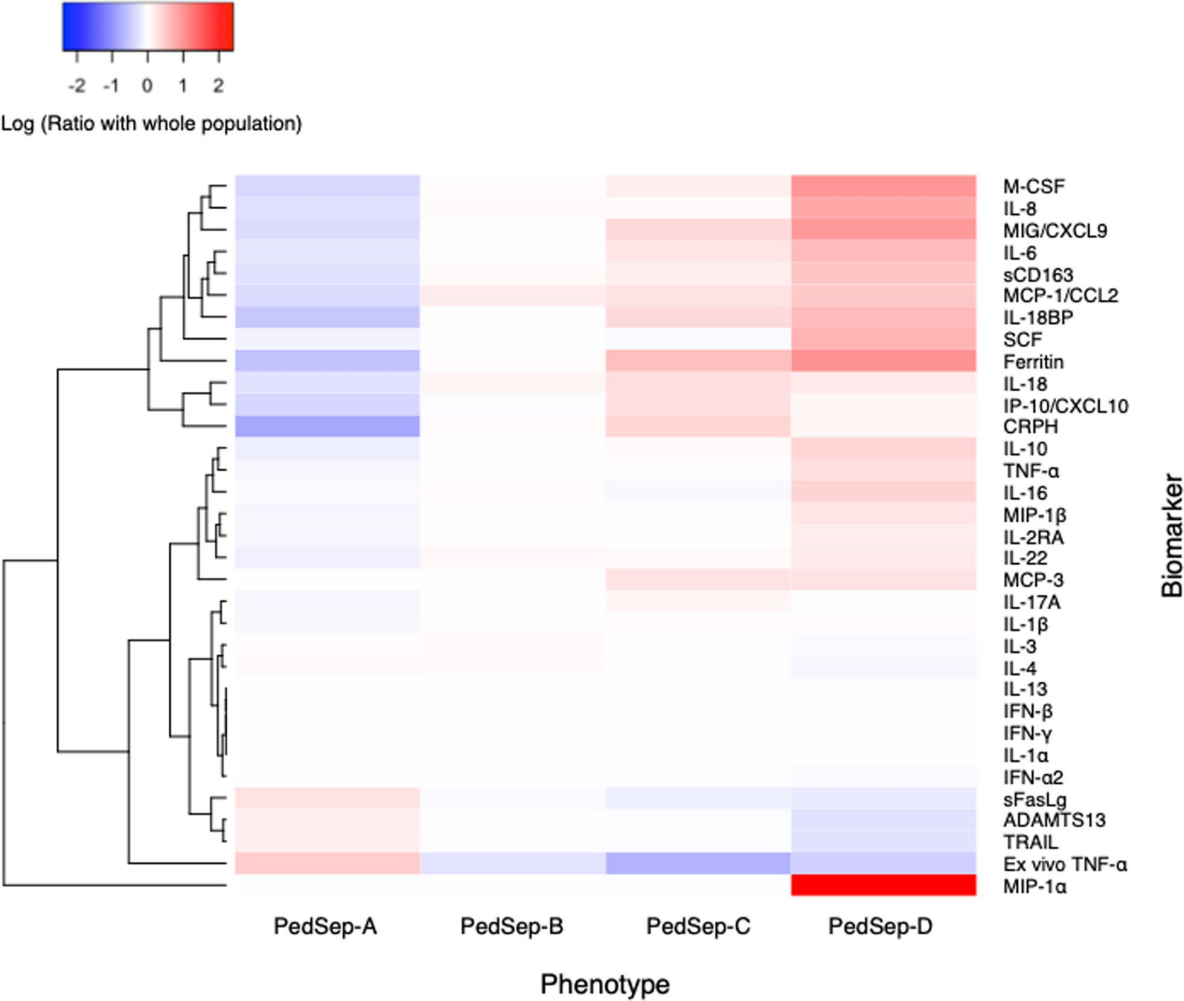
Ratio of inflammatory biomarkers according to 24-h phenotypes. The cytokine heatmap shows the log ratio of the median biomarker values for various markers of the host response and their hierarchical cluster relationships. Red represents a greater median biomarker value for that phenotype compared with the median for the entire study cohort, whereas blue represents a lower median biomarker value compared with the median for the entire study cohort. For example, M-CSF is lower in PedSep-A than the entire study cohort and is higher in PedSep-D than the entire study cohort

**Fig. 4:**
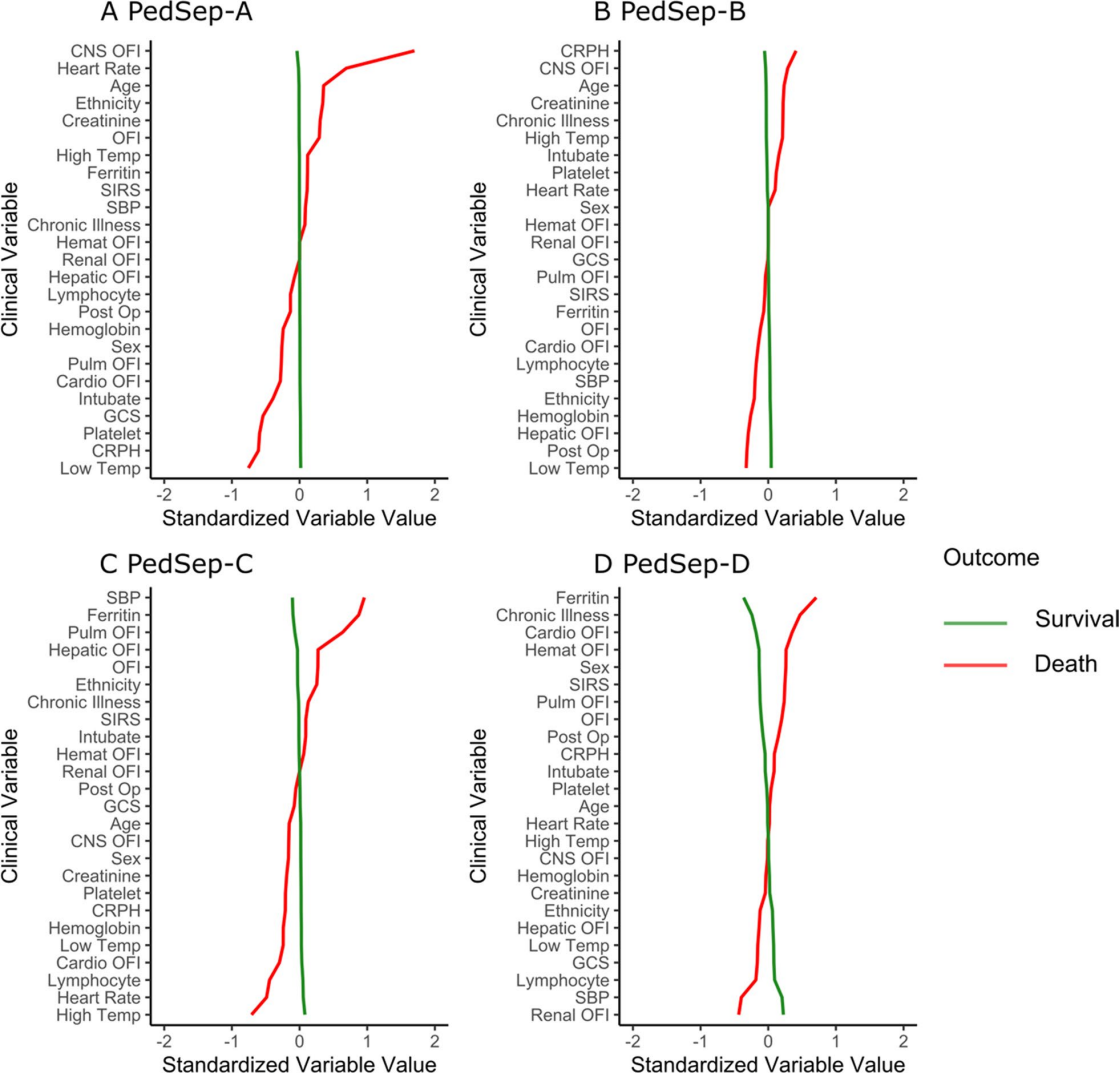
Comparison of relationships of 25 variables to mortality in PedSep-A, B, C, and D. In all panels, the variables are standardized such that all means are scaled to 0 and SDs to 1. A value of 1 for the standardized variable value (*x*-axis) signifies that the mean value for the phenotype was 1 SD higher, or lower for − 1, than the mean value for the phenotypes shown in the graph as a whole. *CNS* central nervous system, *CRPH* C-reactive protein, *GCS* Glasgow Coma Scale, *Hemat* hematologic, *Intubate* intubation with endotracheal tube, *OFI* organ failure index, *Post-Op* post-surgery, *Pulm* pulmonary, *Temp* temperature, *SBP* systolic blood pressure, *Chronic illness* those who are not recorded as previous healthy, *Ethnicity* value is higher with more non-Hispanics in group, *Sex* value is higher with more males in group

**Fig. 5:**
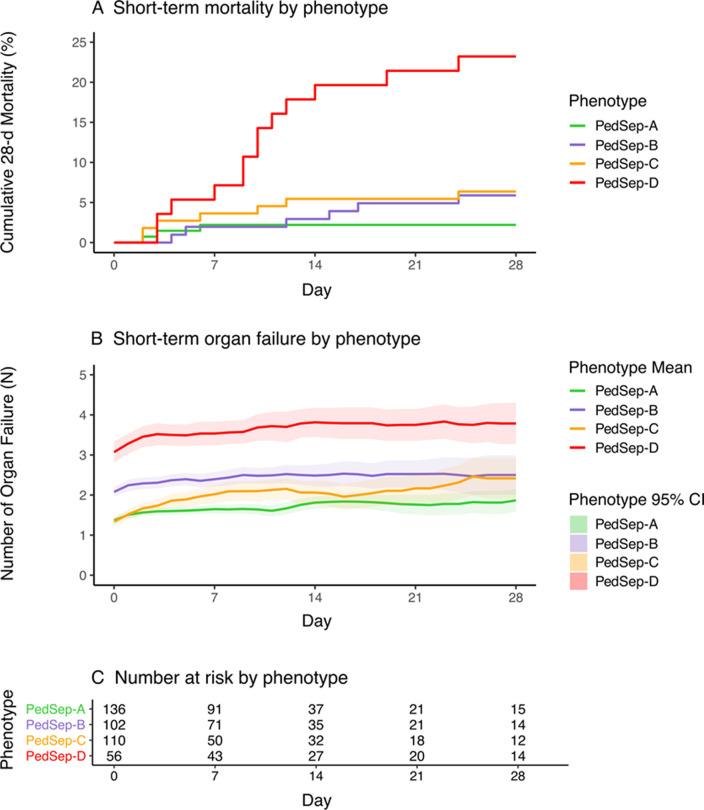
Organ failure and mortality curves over 28 days among 24-h phenotypes. Number of organ failures and mortality according to PedSep-A, B, C, and D phenotype over 28 days. Both short-term mortality (panel **A**) and organ failure (panel **B**) show significant differences by phenotype (*p* < 0.001). The mean numbers of organ failures and 95% confidence intervals (CI) are calculated each day by non-nested observation, where we do not carry forward the OFI at the time the patient leaves the PICU alive or dead. As a reference for patients at risk for Panel **B**, Panel **C** shows the number of children remaining in the PCU at day 0, 7, 14, 21, and 28

DeMerle et al. suggested that derived machine learning phenotypes need to be *clinically relevant, biologically plausible, nonsynonymous, treatment responsive,* and *reproducible* if they are to provide a *‘path forward’* in trial design [25]. Our statistical approach toward these goals is shown in Fig. 1. To derive *nonsynonymous* computable phenotypes, we applied unsupervised clustering methods [26] to a priori clinical and laboratory data available at the first 24 h of PICU stay with severe sepsis (Table 1, Fig. 2, Additional file 1: Tables S1–S4, Additional file 1: Figs. S2–S7). To understand *biological plausibility and clinical relevance*, we examined correlations between the derived computable phenotypes and the a priori diagnoses, infections, and inflammatory cytokine responses (Table 2, Fig. 3, Additional file 1: Tables S5–S8, Additional file 1: Figs. S8–S10), and a priori organ failure and mortality outcomes (Table 2, Figs. 4 and 5, Additional file 1: Tables S9–S11, Additional file 1: Figs. S11–S15). We further examined correlations between the derived computable phenotypes and the a priori elements immunoparalysis-associated MOF (immune depression defined by ex vivo TNF response to endotoxin < 200 pg/ml beyond three days with two or more organ failures) [8, 15, 27], thrombocytopenia-associated MOF (thrombotic microangiopathy defined by ADAMTS13 activity < 57% of control with platelet count < 100,000/mm^3^ and acute kidney injury with oliguria and serum creatinine > 1 mg/dL) [9, 15, 27], sequential liver failure-associated MOF (lymphoproliferative disease associated with liver failure defined by soluble FAS ligand > 200 pg/mL with PaO2/FiO2 < 300 and mechanical ventilation followed seven days or later with serum ALT > 100 U/L and bilirubin > 1 mg/dL) [11, 15, 27], and macrophage activation syndrome (hyperinflammation defined by ferritin > 500 ng/mL with platelet count < 100 K/mm^3^, INR > 1.5, ALT > 100 U/L and bilirubin > 1 mg/dL) (Table 2, Additional file 1: Table S9, Additional file 1: Fig. S14) [12, 13, 15, 27].

**Table 1:** Demographic and day 1 clinical characteristics of the four phenotypes

Characteristic^1^	Total	PedSep-A	PedSep-B	PedSep-C	PedSep-D
No. of patients, *N* (%)	404 (100)	136 (34)	102 (25)	110 (27)	56 (14)
Demographic					
Age years* mean (SD)	7 (6)	3 (4)	8 (6)^a^	10 (5)^a,b^	8 (6)^a^
Male* *N* (%)	224 (55.4)	63 (46.3)	68 (66.7)^a^	59 (53.6)	34 (60.7)
Female* *N* (%)	180 (44.6)	73 (53.7)	34 (33.3)	51 (46.4)	22 (39.3)
Hispanic* *N* (%)	67 (16.6)	28 (20.6)	12 (11.8)	23 (20.9)	4 (7.1)
Non-Hispanic* *N* (%)	323 (80.0)	100 (73.5)	86 (84.3)	86 (78.2)	51 (91.1)
Previous healthy* *N* (%)	180 (44.6)	96 (70.6)^b,c,d^	28 (27.5)	37 (33.6)	19 (33.9)
Surgery* *N* (%)	49 (12.1)	6 (4.4)	19 (18.6)^a^	12 (10.9)	12 (21.4)^a^
Organ dysfunction					
SIRS criteria, mean (SD)^2^	2.9 (0.8)	2.9 (0.8)	3.0 (0.8)	2.8 (0.8)	3 (0.8)
OFI* mean (SD)^3^	1.8 (0.9)	1.4 (0.5)	2.1 (0.6)^a,c^	1.4 (0.6)	3.1 (1.0)^a,b,c^
Inflammation					
CRP mg/dL* mean (SD)	11.7 (10.4)	7.3 (7.3)	13.2 (11.5)^a^	15.2 (10.4)^a^	13.1 (11.2)^a^
Low temperature °C* mean	36.6 (1.2)	36.7 (0.9)^b^	36.0 (1.6)	37.1 (0.9)^a,b,d^	36.3 (1.0)
High temperature °C* mean	37.8 (1.3)	37.8 (1.1)	37.4 (1.3)	38.3 (1.2)^a,b,d^	37.8 (1.4)
ALC/mm^3^* median (IQR)	1.2 (0.6–2.1)	1.9(1.3–3.2)^b,c,d^	1.1(0.6–1.9)^c^	0.6 (0.2–1.0)	1.1(0.6–2.1)^c^
Ferritin ng/mL* median (IQR)	218 (98.0–625.3)	125(69.8–207.8)	223(116.5–544.2)^a^	405(176.2–1485.7)^a,b^	610 (221.1–2482.0)^a,b^
Pulmonary					
Pulmonary OFI* *N* (%)	270 (66.8)	108 (79.4)^c^	87 (85.3)^c^	37 (33.6)	38 (67.9)^c^
Intubation* *N* (%)	211 (52.2)	72 (52.9)^c^	94 (92.2)^a,c,d^	15 (13.6)	30 (53.6)^c^
Cardiovascular or hemodynamic					
Heart rate bpm* mean (SD)	155.4 (31.3)	168.1 (30.8)^b,c,d^	146.5 (27.9)	150.4 (27.6)	150.6 (35.8)
Systolic blood pressure*, mean (SD) mmHg	81.9 (19.3)	85.0 (15.7)^b^	74.8 (22.0)	86.3 (17.2)^b^	78.9 (21.9)
Cardiovascular OFI* *N* (%)	284 (70.3)	63 (46.3)	92 (90.2)^a^	85 (77.3)^a^	44 (78.6)^a^
Renal					
Creatinine mg/dL* median (IQR)	0.5 (0.3–0.8)	0.3 (0.2–0.4)	0.6 (0.4–1.0)^a^	0.6 (0.4–0.7)^a^	1.4 (0.6–2.6)^a,b,c^
Renal OFI* *N* (%)	30 (7.4)	0 (0.0)	0 (0.0)	0 (0.0)	30 (53.6)^a,b,c^
Hepatic					
Hepatic OFI* *N* (%)	40 (9.9)	3 (2.2)	9 (8.8)	11 (10.0)^a^	17 (30.4)^a,b,c^
Hematologic					
Hemoglobin g/dL* mean (SD)	9.8 (2.0)	10.1 (1.8)^b,d^	9.4 (2.1)	10.2 (2.1)^b,d^	9.1 (1.8)
Platelets K/mm^3^* mean (SD)	171.1 (123.2)	260.1 (122.0)^b,c,d^	154.3 (95.1)^c,d^	118.8 (83.5)^d^	88.2 (108.0)
Hematologic OFI* *N* (%)	39 (9.7)	0 (0.0)	0 (0.0)	8 (7.3)^a,b^	31 (85.7)^a,b,c^
Neurologic					
Glasgow Coma Scale Score* mean (SD)^4,5^	8.7 (5.3)	8.5 (5.2)^b^	4.7 (3.4)	13.2 (3.1)^a,b,d^	7.9 (5.5)^b^
CNS OFI *N* (%)	54 (13.4)	12 (8.8)	24 (23.5)^a,c^	6 (5.5)	12 (21.4)^c^

*IQR* interquartile range, *SIRS* systemic inflammatory response syndrome, *OFI* organ failure index, *ALC* absolute lymphocyte count, *CNS* central nervous system

SI conversion factors: to convert alanine transaminase and aspartate aminotransferase to μkat/L, multiply by 0.0167; bilirubin to μmol/L, multiply by 17.104; C-reactive protein to nmol/L, multiply by 9.524; creatinine to μmol/L, multiply by 88.4

*Comparisons across all 4 computable phenotypes were performed using the Kruskal–Wallis test, the *χ*^2^ test, or the Fisher’s exact test (Additional file 1: Table S3, *p* < 0.05 for all comparisons after adjustment)

^1^The variables in this table were log transformed for modeling (Additional file 1: Table S3). Comparisons across all 4 phenotypes were performed using the Kruskal–Wallis test, the *χ*^2^ test, or the Fisher’s exact test (Additional file 1: Table S3. *p* < 0.05 for all comparisons after adjustment)

^2^Indicates SIRS criteria ranging from 0 to 4 including abnormal heart rate, respiratory rate, temperature, and white blood cell count

^3^OFI is an integer score reflecting the number of organ failures. Scores are either 0 or 1 for cardiovascular, hepatic, hematologic, respiratory, neurological, and renal, and summed for total range of 0 to 6. Cardiovascular, need for cardiovascular agent infusion support; Pulmonary, need for mechanical ventilation support with the ratio of the arterial partial pressure of oxygen and the fraction of inspired oxygen (PaO_2_/FiO_2_) < 300 without this support; Hepatic, total bilirubin > 1.0 mg/dL and alanine aminotransferase (ALT) > 100 units/L; Renal, serum creatinine > 1.0 mg/dL and oliguria (urine output < 0.5 mL/kg/h); Hematologic, thrombocytopenia < 100,000/mm^3^ and prothrombin time INR > 1.5 × normal; Central Nervous System, Glasgow Coma Scale (GCS) Score < 12 in the absence of sedatives

^4^Corresponds to minimum or maximum value (as appropriate) within 6 h of hospital presentation

^5^GCS ranges from 3 to 15

^a^The outcome characteristic of this computable phenotype is significantly higher than PedSep-A (*p* value < 0.05)

^b^The outcome characteristic of this computable phenotype is significantly higher than PedSep-B (*p* value < 0.05)

^c^The outcome characteristic of this computable phenotype is significantly higher than PedSep-C (*p* value < 0.05)

^d^The outcome characteristic of this computable phenotype is significantly higher than PedSep-D (*p* value < 0.05)

**Table 2:** Subsequent outcome characteristics of the four phenotypes

Characteristic^e^	Total	PedSep-A	PedSep-B	PedSep-C	PedSep-D
No. of patients, *N* (%)	404 (100)	136 (34)	102 (25)	110 (27)	56 (14)
Development of subsequent MOF empirical phenotypes					
SMOF, *N* (%)	7 (1.7)	0 (0.0)	0 (0.0)	1 (0.9)	6 (10.7)^a,b,c^
TAMOF, *N* (%)	37 (9.2)	0 (0.0)	6 (5.9)^a^	3 (2.7)	28 (50.0)^a,b,c^
IPMOF, *N* (%)	85 (21.0)	12 (8.8)	29 (28.4)^a^	22 (20)	22 (39.3)^a^
MAS, *N* (%)	24 (5.5)	0 (0.0)	3 (2.9)	2 (1.8)	19 (33.9)^a,b,c^
NPMOF, *N* (%)	117 (29.0)	28 (20.6)	25 (24.5)	32 (29.1)	32 (57.1)^a,b,c^
Infections					
Bacterial infection, *N* (%)	141 (34.9)	43 (31.6)	33 (32.4)	45 (40.9)	20 (35.7)
Viral infection, *N* (%)	114 (28.2)	60 (44.1)^b,c,d^	21 (20.6)	24 (21.8)	9 (16.1)
Fungal infection, *N* (%)	4 (1.0)	0 (0.0)	1 (1.0)	0 (0.0)	3 (5.4)
Culture negative, *N* (%)	177 (43.8)	47 (34.6)	52 (51.0)	50 (45.5)	28 (50.0)
Sites of infections^f^					
Blood, *N* (%)	51 (12.6)	10 (7.4)	6 (5.9)	22 (20.0)^a,b^	13 (23.2)^a,b^
Lung, *N* (%)	76 (18.8)	28 (20.6)	29 (28.4) ^a,c,d^	12 (10.9)	7 (12.5)
Urine, *N* (%)	16 (4.0)	4 (2.9)	5 (4.9)	6 (5.5)	1 (1.8)
Organ support					
MechVent, *N* (%)	366 (90.6)	134 (98.5)^c^	101 (99.0)^c^	79 (71.8)	52 (92.9)^c^
ECMO, *N* (%)	30 (7.4)	5 (3.7)	9 (8.8)	6 (5.5)	10 (17.9)^a^
CRRT, *N* (%)	52 (12.9)	1 (0.7)	7 (6.9)	7 (6.4)	37 (66.1)^a,b,c^
Anti-inflammatory therapies of interest					
Decadron, *N* (%)	94 (23.3)	50 (36.8)^c,d^	22 (21.6)	14 (12.7)	8 (14.3)
Methylprednisolone, *N* (%)	117 (29.0)	54 (39.7)^b^	23 (22.5)	24 (21.8)	16 (28.6)
IVIG, *N* (%)	51 (12.6)	6 (4.4)	10 (9.8)	19 (17.3)^a^	16 (28.6)^a^
IVIG + Methylprednisolone	23 (5.7)	3 (2.2)	4 (3.9)	9 (8.2)^a^	7 (12.5)^a^
Plasma exchange, *N* (%)	25 (6.2)	5 (3.7)	4 (3.9)	4 (3.6)	12 (21.4)^a,b,c^
Plasma exchange + ECMO	6 (1.5)	1 (0.7)	1 (1.0)	1 (0.9)	3 (5.4)
Outcome					
Length of stay, median (IQR), d	9.0 (5.0–17.)	9.0 (5.8–15)^c^	10.5 (5.3–17)^c^	6 (2.3–15)	12.5 (7–26.5)^c^
Mortality, *N* (%)	45 (11.1)	3 (2.2)	12 (11.7)^a^	11 (10.0)^a^	19 (33.9)^a,b,c^
PICU free days, median (IQR), d	20.0 (8.0–25.0)	21.0 (14.8–24.0)^d^	19.0 (9.8–24.0)^d^	24.0 (13.3–27)^a,b,d^	4.5 (0.0–21.0)

*SMOF* sequential liver failure-associated multiple organ failure, *TAMOF* thrombocytopenia-associated multiple organ failure, *IPMOF* immunoparalysis-associated multiple organ failure, *MAS* Macrophage Activation Syndrome, *NPMOF* new or progressive multiple organ failure, *IQR* interquartile range, *MechVent* mechanical ventilation, *ECMO* extracorporeal membrane oxygenation, *CRRT* continuous renal replacement therapies, *IVIG* intravenous gamma globulin

^a^The outcome characteristic of this computable phenotype is significantly higher than PedSep-A (*p* value < 0.05)

^b^The outcome characteristic of this computable phenotype is significantly higher than PedSep-B (*p* value < 0.05)

^c^The outcome characteristic of this computable phenotype is significantly higher than PedSep-C (*p* value < 0.05)

^d^The outcome characteristic of this computable phenotype is significantly higher than PedSep-D (*p* value < 0.05)

^e^Comparisons across all 4 computable phenotypes were performed using the Kruskal–Wallis test, the *χ*^2^ test, or the Fisher’s exact test (Additional file 1: Table S3, *p* < .05 for all comparisons after adjustment)

^f^Obtained at the first 3 days

**Fig. 6:**
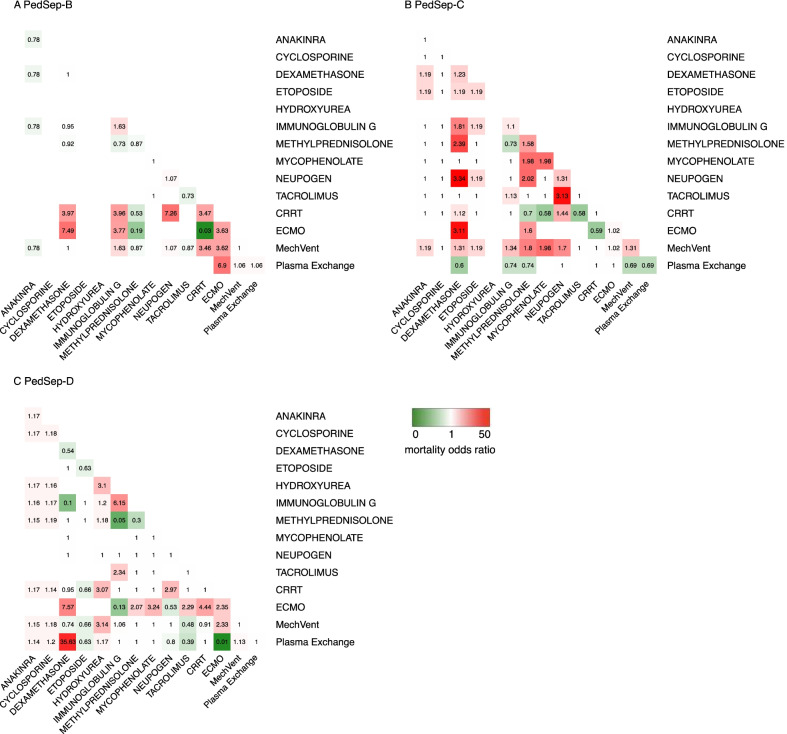
Heterogeneous treatment interactions and mortality risks among the phenotypes. Heatmap of Elastic Net Regression analysis shows the association between 14 individual therapies (diagonal values) and their 91 combination interactions (total cells = 105) with mortality in PedSep-B, C, and D among children who received anti-inflammatory therapies. The PedSep-A phenotype is not presented due to limited number of deaths. Blank cells have no patients. Values in each cell represent odds ratios of mortality, where 1 represents no association with mortality. Color in each cell represents direction of effect, where red represents mortality direction, green represents survival direction. Cells located at the diagonal are odds ratio of association from the 14 individual therapies. The other cells represent the mortality odds ratio of combinations of these therapies compared to all other combinations. For example, survivors in PedSep-D phenotype are less likely to be treated with IVIG than non-survivors (red), whereas survivors in PedSep-D are more likely to be treated with combined IVIG + methylprednisolone (green). This machine analysis method does not allow calculation of confidence intervals

In exploratory preliminary analysis of a priori anti-inflammatory treatments, we assessed interactions with one another among the derived phenotypes in patients who received any of these anti-inflammatory therapies. We applied elastic net regression analysis to any a priori organ support and anti-inflammatory therapies used by bedside clinicians that were found in univariable analysis to be associated with survival in any of the derived computable phenotypes or in the population as a whole (*p* < 0.05) (Fig. 6, Additional file 1: Tables S12 and S13, Additional file 1: Fig. S15) [28]. Because elastic net regression analysis does not allow for calculation of 95% confidence intervals, we further applied logistic regression analysis to any anti-inflammatory therapy interactions associated with mortality odds ratio < 0.1 in the elastic net regression model (Additional file 1: Tables S14–S17).

### Candidate clinical variables for phenotyping

Of the 52 bedside variables collected a priori in the parent study, only 25 were available at 24 h with less than 20% missingness and less than 60% correlation with any other variable (Table 1, Additional file 1: Tables S1 and S2, Additional file 1: Fig. S2). These included demographic variables (age, gender, ethnicity, previous health status, post-op status), PRISM-related vital signs and laboratory values (systolic blood pressure, heart rate, Glasgow Coma Scale Score, hemoglobin, creatinine, platelet count, intubation status), markers of inflammation (temperature, number of SIRS criteria, lymphocyte count, C-reactive protein level, ferritin level), and organ failures (Central Nervous System = Glasgow Coma Scale < 12 not explained by use of sedation; Cardiovascular = Requirement for vasoactive agents for Systolic Blood Pressure < 5th percentile for age; Respiratory = PaO_2_/FiO_2_ ratio < 300 requiring mechanical ventilation; Renal = oliguria and serum creatinine > 1 mg/dL; Hepatic = ALT > 100 and Bilirubin > 1 mg / dL; Hematologic = Platelet Count < 100 K and INR > 1.5) [11, 15, 27]. For each a priori PRISM variable we extracted the most abnormal value in the first 6 h. For each a priori inflammation and organ failure variable we extracted the most abnormal value within 24 h. Consensus k-means clustering models were used to derive 24-h computable phenotypes using these 25 variables because the method provides nonsynonymous agnostic clusters and has a 1000 iterations step to assure internal consistency [20].

Specific chronic illnesses and present illnesses were not included in the derived 25-element phenotype assessment model. Specific sites of infection (blood, lung, urine) and etiologies of infection (bacterial, fungal, viral, and culture negative) were not included in the derived 25 element phenotype assessment model because they were not reliably available in the first 24 h.

### Biological correlates and outcomes

We studied 33 a priori biomarkers including 31 cytokines and two functional assays concomitantly measured one day only, at day 1 of severe sepsis; whole blood ex vivo TNF response to endotoxin as a marker of immune depression [7, 8, 15, 27], and ADAMTS 13 activity as a marker of microvascular thrombosis in the presence of thrombocytopenia [7, 15, 27]. Plasma for cytokine measurement was divided into three assays. IL-18, IL-18BP, and CXCL9 were measured at 25-fold dilution [29]. IFN α, sCD163, and IL-22 were measured by Bioplex inflammatory flex-set assay per manufacturer’s instructions (Bio-Rad). The remainder were measured by Bioplex Group I/II flex-set assay (Bio-Rad). All cytokines were measured on a BioPlex 200 System (Bio-Rad). The functional assays were measured as previously described [7–9, 15, 27].

The a priori primary outcome was hospital mortality. The a priori secondary outcomes included development of new or progressive MOF defined as development of new organ failure(s) after day one [2]; PICU free days at 30 days with 0 days given for death; length of stay in the PICU; development of immunoparalysis [8, 9, 15, 27], thrombocytopenia-associated MOF [9, 15, 27], sequential liver failure-associated MOF [11, 15, 27], and macrophage activation syndrome [12, 13, 15, 27]; as well as use of mechanical ventilation, and extracorporeal therapies.

Derived elastic net regression results are presented as unadjusted odds ratios. All other derived odds ratio analyses and interactions are presented are adjusted controlling for age, sex, ethnicity, race, and total PRISM score. For summary analyses, the threshold for statistical significance was less than 0.05 for two-sided tests after adjustment for multiple testing. All analyses were performed with R version 3.6.2.

## Results

### Derivation of clinical sepsis phenotypes

The derived consensus *k*-means clustering models [20] found a 4-class model was the optimal fit, with phenotypes we named PedSep-A, B, C and D (Additional file 1: Figs. S3 and S4). Consensus matrix plots and the relative change under cumulative distribution function curve implied little statistical gain by increasing to a 5 or 6 class model, with penalty of overfitting. The size and characteristics of the 4-class model are given in Table 1 and Fig. 2. They ranged in size (from 14 to 34% of the cohort) and differed in clinical characteristics and organ dysfunction patterns (Table 1, Additional file 1: Table S3, Fig. 2, Additional file 1: Figs. S5 and S11). With the exception of the SIRS criteria number, all of the other 24 variables differed among the phenotypes. Compared to all other phenotypes, PedSep-A patients were younger and previously healthy, with the lowest CRP and ferritin levels, the highest lymphocyte and platelet counts, highest heart rate, and lowest creatinine; PedSep-B patients were most likely to be intubated and had the lowest Glasgow Coma Scale Score; PedSep-C patients had the highest temperature and Glasgow Coma Scale Score, least pulmonary failure, and lowest lymphocyte count; and PedSep-D patients had the highest creatinine and number of organ failures, including renal, hepatic, and hematologic organ failure, with the lowest platelet count. On average, PedSep-B and D patients had multiple organ failure, whereas PedSep-A and C patients did not. Ferritin levels were highest in PedSep-C and PedSep-D distinguishing them from PedSep-A and B (Table 1, Additional file 1: Table S3, Fig. 2, Additional file 1: Figs. S5 and S11).

### Correlation of phenotypes with diagnoses

Differences were noted among the derived phenotypes in diagnoses including leukemia (PedSep-D and C > PedSep-A and B; PedSep-B > PedSep-A), inflammatory bowel disease (PedSep-D > PedSep-A and B), chromosomal abnormality (PedSep-D > PedSep-C), metabolic disease (PedSep-B > PedSep-C and D), cardiovascular disease + postoperative status (PedSep-D > PedSep-C), short gut syndrome (PedSep-C > PedSep-A), and acute bronchiolitis (PedSep-A > PedSep-B and C) (Additional file 1: Tables S5 and S6, Additional file 1: Fig. S8). There were no differences noted among the derived phenotypes in the diagnoses of hemolytic anemia, rheumatic disease, renal disease, diabetes, cardiovascular disease, trauma, or liver disease.

### Correlation of phenotypes with biomarker profiles

The inflammatory biomarker profiles differed across the four derived computable phenotypes. Inflammation evidenced by cytokine signature increased, and immune response (whole blood ex vivo TNF response to endotoxin) and coagulation function (ADAMTS13 activity) decreased going across PedSep-A, B, C, and D (Additional file 1: Tables S7 and S8, Fig. 3, Additional file 1: Fig. S10). PedSep-A showed the least inflammation with the lowest M-CSF, IL-8, IL-6, sCD163, MCP1/CCL2, ferritin, C-reactive protein, IL-10, IL-22, IL-18, IL-18BP, and MIP 1α levels overall; lower CXCL9 than PedSep-C and D; lower IL-17a than PedSep-B and C; lower IP10/CXCL10 than PedSep-C; and lower IL2Ra than PedSep-D. PedSep-A had the best immune and coagulation function with normal whole blood ex vivo TNF response to endotoxin (> 200 pg/mL) and ADAMTS 13 activity. In contrast, PedSep-D had the most profound inflammatory response with highest M-CSF, IL-8, SCF, sCD163, IL-16, IL-10, TNF, and MIP1α levels, and thrombotic microangiopathic response with lowest ADAMTS13 activity decreased to < 57% of control with thrombocytopenia. Consistent with this increased inflammation response, the macrophage inhibitor TRAIL was reduced in PedSep-D compared to PedSep-C. PedSep-D also had higher CXCL9 then PedSep-B but not PedSep-C.

### Relationship with infection, organ support needs, and hospital mortality

PedSep-A had more viral infections, PedSep-B had more pneumonia, and PedSep-C and D had more blood infections (Table 2, Additional file 1: Table S9, Additional file 1: Fig. S9). Patients in PedSep-C had the least mechanical ventilation and the shortest length of stay. Patients in PedSep-D required more extracorporeal membrane oxygenation than in PedSep-A, and the most continuous renal replacement therapy (CRRT) overall. PedSep-A patients required the least CRRT. PICU free days were highest in PedSep-C and lowest in PedSep-D (Table 2, Additional file 1: Table S9).

Hospital mortality was 2% in PedSep-A, 12% in PedSep-B, 10% in PedSep-C, and 34% in PedSep-D (PedSep-B vs. A Adj OR 4.11 95% CI [1.11–19.96] *p* = 0.048; PedSep-C vs. A Adj OR 4.35 95% CI [1.23–20.43] *p* = 0.034; PedSep-D vs. A Adj OR 17.25 95% CI [4.93–92.06] *p* = 4.42E−05; PedSep-D vs B Adj OR 4.20 95% CI [1.84–9.97] *p* = 0.0008; and PedSep-D vs. C Adj OR 3.97 95% CI 1.62–10.14] *p* = 0.003) (Table 2, Additional file 1: Table S9).

The derived mortality curves show all deaths in PedSep-A occurred before seven days, whereas deaths in PedSep-B, C, and D continued to accrue after seven days (Fig. 5, Additional file 1: Fig. S13). Mortality was associated with Glasgow Coma Scale Score < 12, decreased TNF and IL-2Ra levels, and increased MCP3 levels in PedSep-A; increased IL-6, IL-8, and MCP1/CCL2 levels in PedSep-B; high ferritin, lymphopenia, lower temperature, higher blood pressure, and increased IL-8 levels in PedSep-C; and hyperferritinemia, chronic illness, increased MIP-1α, IL-8, and IL-10 levels, and decreased IL-18 and sFASL levels in PedSep-D (Fig. 4, Additional file 1: Tables S9–S11, Additional file 1: Fig. S12).

### Relationship with development of immunoparalysis, TAMOF, SMOF, and MAS

On average, children in PedSep-A and PedSep-C developed less than two organ failures; children in PedSep-B developed more than two organ failures; and children in PedSep-D developed more than three organ failures over 28 days (Fig. 5, Additional file 1: Fig. S13). Children in PedSep-D had the highest proclivity to develop immunoparalysis (Adj OR 2.40 95% CI [1.25–4.53; *p* = 7.20E-03), new and progressive organ failure (Adj OR 4.03 95% CI [2.19–7.55]; *p* = 9.48E-06), thrombocytopenia-associated MOF (Adj OR 47.51 95% CI [18.83–136.83]; *p* = 1.25E-14), sequential liver failure-associated MOF (Adj OR 61.56 95% CI [8.93–1,282.58]; *p* = 3.80E-04), and macrophage activation syndrome (Adj OR 38.63 95% CI [13.26–137.75]; *p* = 4.61E-10). Immunoparalysis- and thrombocytopenia-associated MOF also occurred more commonly in children in PedSep-B and D compared to those in PedSep-A (Table 2, Additional file 1: Fig. S14).

### Heterogeneous treatment interactions with therapies

All 3 organ support therapies and 11 of 41 anti-inflammatory therapies were associated with outcome in univariable analysis (Additional file 1: Tables S12 and S13, Additional file 1: Fig. S15) among the children who received anti-inflammatory therapies and were included in the derived exploratory elastic net regression analysis (Fig. 6, Additional file 1: Fig. S15) [28]. This was not performed in PedSep-A because mortality was very low at 2%. The constructed elastic net regression heatmaps visualize heterogeneous mortality association patterns across PedSep-B, C, and D (Fig. 6). Unadjusted mortality odds ratios < 0.1 with use of anti-inflammatory agents were not observed with any single therapy; however, unadjusted interactions < 0.1 were observed with use of methylprednisolone and IVIG together, and in extracorporeal membrane oxygenator patients receiving plasma exchange, in PedSep-D (Fig. 6).

Combined use of methylprednisolone plus IVIG was more common in PedSep-C and D than in A and B (Table 2). Methylprednisolone was administered on median day 1 (25th–75th % tile days 1–3) for a median duration of 5 days (25th–75th % tile 2–7 days). IVIG was administered on median day 2 (25th–75th% tile day 1–7) for a median duration of 1 day (25th-75th % tile 1–3 days) (Additional file 1: Table S13). Neither methylprednisolone nor IVIG treatment alone, nor the combination, was associated with reduced odds of mortality in adjusted logistic regression modeling (Additional file 1: Table S14, S15, and S16). The interaction term < 0.1 identified in elastic net regression analysis between methylprednisolone and IVIG therapies in PedSep-D patients remained statistically significant in logistic regression analysis (Methylprednisolone * IVIG interaction = 0.03; 95% CI [0.00058–0.66] *p* = 0.04) interpreted as meaning that the association of IVIG with mortality was modified by exposure to methylprednisolone in PedSep-D patients (Additional file 1: Tables S14 and S15). There was also a significant interaction between PedSep-D membership and use of combined methylprednisolone plus IVIG therapy in logistic regression analysis (PedSep-D * Methylprednisolone + IVIG combination interaction = 0.04 95% CI [0.001–0.56] *p* = 0.026) interpreted as meaning that the mortality association with exposure to combined methylprednisolone plus IVIG use is modified by PedSep-D membership (Additional file 1: Tables S16 and S17). Plasma exchange was most commonly used in PedSep-D (Table 2). The interaction term < 0.1 identified in elastic net regression modeling between ECMO and plasma exchange use was not statistically significant in logistic regression modeling (ECMO * plasma exchange interaction = 0.02 95% CI [0.000165–0.97] *p* = 0.06) (Additional file 1: Table S15).

## Discussion

Machine learning analysis of a priori elements from the extant PHENOMS study derived four computable 24-h phenotypes meeting three of five ‘path forward’ criteria [25] providing impetus for their further evaluation in new pediatric sepsis studies. The derived computable phenotypes demonstrated *clinical relevance* with differences in types of presenting diagnoses, infections, organ failures, need for organ support therapies, outcomes, and proclivity to development of TAMOF and MAS. Derived consensus *k*-means clustering and t-SNE analyses demonstrated that the computable phenotypes are *nonsynonymous.* The differences in cytokine profiles provide *biological plausibility* for these derived computable phenotypes having different inflammation responses, highlighted in PedSep-D by decreased ADAMTS13 with TAMOF and increased MIP 1α with MAS. Exploratory modeling of interactions between therapies among patients receiving anti-inflammatory treatments, derived computable phenotypes, and mortality demonstrated no reduction in mortality odds with methylprednisolone, IVIG or the combination; however, it identified a signal for methylprednisolone affecting the relationship of IVIG therapy to outcome in PedSep-D patients. We speculate that this interaction is reminiscent of the report that addition of methylprednisolone to IVIG improves cardiac function in children with COVID19-related multisystem inflammatory syndrome (MIS-C) compared to IVIG alone [30]. The very wide confidence intervals provide impetus to further evaluate this interaction signal in larger sample sizes using new study cohorts. We are presently assessing *treatment responsiveness* and *reproducibility* of the four derived phenotypes in our NICHD network’s 1000 patient *Personalized Immunomodulation in Pediatric Sepsis and Multiple Organ Dysfunction* trial testing interleukin 1 antagonist protein for hyper-inflammatory sepsis; and, also in the observational 500 patient *Second Argentinian Pediatric Sepsis Epidemiology Study* (PI Roberto Jabornisky).

PedSep-A is characterized by younger previously healthy children with respiratory failure and the least increased inflammation. This resembles the adult α phenotype in the SENECA trial [20], and also the MARS 3 and sepsis response signature 2 endotypes, which found predominant expression of adaptive immune and B-cell developmental pathways [31–33]. Mortality in PedSep-A was low at 2% and did not increase after 7 days, making anti-inflammatory clinical trials directed to survival less feasible.

PedSep-B is characterized by multiple organ failure requiring intubation for more severe respiratory failure, shock, and central nervous system dysfunction with increased C-reactive protein levels and 12% mortality. This is reminiscent of children reported in the *Life After Pediatric Sepsis Evaluation* study [34]; the shock with hypoxia phenotype in adult sepsis-induced MOF [35]; and the severe hypoxia, altered mental status, and shock phenotype in pediatric MOF [36].

PedSep-C is distinguished by cardiovascular failure and relative absence of need for intubation (14%) with the least pulmonary failure (34%) and need for mechanical ventilation (71%), in the presence of elevated C-reactive protein, high ferritin, and lymphopenia, with 10% mortality. This is reminiscent of the Toxic Shock (TSS)—Kawasaki syndrome phenotype currently being considered as PMIS/MIS-C syndrome [30, 37–40]. Similar to TSS and Kawasaki’s, our PedSep-C patients showed elevated IL-17a and IP10/CXCL10 levels [41, 42].

PedSep-D patients had cardiovascular, respiratory, liver, renal, hematologic, and neurologic dysfunction with 34% mortality; clinical features shared by the adult δ phenotype characterized in the SENECA study using electronic health record criteria for Sepsis-3 [20]; the shock with thrombocytopenia pediatric MOF phenotype [36]; and previously reported subclasses including the hyperinflammatory sub-phenotype reported in acute respiratory distress syndrome, a condition commonly related to sepsis [43–45]. It also resembles sepsis endotypes derived using transcriptomic analyses of circulating immune cells, specifically the inflammopathic cluster known as sepsis signature 1, or the Molecular Diagnosis and Risk Stratification of Sepsis [MARS] 2 cluster [31–33].

PedSep-D is specifically characterized by hyperferritinemic (ferritin > 500 ng/mL), thrombocytopenic (platelet count < 100 K) multiple organ failure with the highest likelihood of new or progressive multiple organ failure accruing mortality after 7 days and the lowest number of PICU free days. PedSep-D membership identifies children with the highest proclivity for decreased ADAMTS 13 activity with thrombocytopenia-associated MOF, and increased MIP 1α with macrophage activation syndrome.

There are limitations to consider in this post hoc machine learning analysis of the parent PHENOMS study and its inherent selection bias risks. Although the PHENOMS study represents the largest longitudinal multiple center pediatric sepsis-induced MOF cohort with concomitant CRP and ferritin levels available [15], it is small compared to adult standards because sepsis occurs 15 times more commonly in adults than in children. Definitions of pediatric sepsis and organ failures are also evolving and behind the changes in adult sepsis. Definitions of sepsis and organ failure were necessarily limited to those used in the extant study. Only 25 out of 52 available clinical and laboratory variables available in this parent study had < 20% missingness without covariance and were included in the machine learning derivation. Only 33 additional biomarkers [8, 9, 11, 27, 45] were performed to assess biological plausibility for the computable phenotypes having different inflammatory responses. Lactate was not recorded and may be an important missing variable [46]. Interactions could only be assessed for those therapies given by bedside clinicians in a ‘natural experiment’ setting. Our models did not capture all confounders, comorbidities, therapies used, reasons for therapies, or site differences in clinical practice. Furthermore, combined methylprednisolone plus IVIG and ECMO plus plasma exchange therapies were rarely administered. Reproducibility of the derived computable phenotypes cannot be assessed in a single extant multiple center resource rich study. We are presently assessing reproducibility in two ongoing independent cohort studies.

## Conclusions

Machine learning analysis in the PHENOMS study derived four novel computable 24-h pediatric sepsis phenotypes providing a computer tool (www.pedsepsis.pitt.edu) that enables clinical researchers to perform bedside identification of an individual patient’s computable phenotype membership. If proven reproducible, then PedSep-D membership appears most optimal for identifying children for early enrollment in personalized anti-inflammatory trials targeting thrombocytopenia-associated MOF and macrophage activation syndrome.

## Supplementary Information

**Additional file 1.** Detailed statistical methods overview.

## Data Availability

All data generated or analyzed during this study are included in this published article [and its supplementary information files]. The datasets used and/or analyzed during the current study are available from the corresponding author on reasonable request. The PHENOMS database is also to be uploaded on the NICHD sponsored DASH website.

## Abbreviations

MOF: Multiple organ failure
PICU: Pediatric Intensive Care Unit
Immunoparalysis MOF, TAMOF: Thrombocytopenia-associated MOF
MAS: Macrophage activation syndrome
SMOF: Sequential MOF
IVIG: Intravenous immune globulin
NIGMS: National Institute’s of General Medical Sciences
PHENOMS: PHENOtyping pediatric sepsis-induced MOF Study
ADAMTS13: A disintegrin and metalloproteinase with a thrombospondin type 1 motif, member 13
PRISM: Pediatric RISk of Mortality
SIRS: Systemic inflammatory response syndrome
CRRT: Continuous renal replacement therapy
ECMO: Extracorporeal membrane oxygenator
NICHD: National Institutes of Child Health and Human Development
