# Supplementary material for: Machine learning derivation of four computable 24-h pediatric sepsis phenotypes to facilitate enrollment in early personalized anti-inflammatory clinical trials
Source: Crit Care. 2022 May 7;26:128. doi: 10.1186/s13054-022-03977-3 (PMC9077858; doi:10.1186/s13054-022-03977-3)
Supplement: Supplementary file 1 — Additional file 1. Detailed statistical methods overview. [file 13054_2022_3977_MOESM1_ESM.docx]

**Supplementary Digital Content Detailed Overview of Statistical Methods**

1. **Variable selection and missing data imputation for input in Consensus k-means clustering**

The workflow of our statistical methods is presented in Figure 1. The *a* priori parent study (CONSORT eFigure 1) collected 6 demographic variables and 46 clinical variables (eTable 1). For derived clustering, we selected variables based on correlation, distribution, and missingness (eTable 2). First, highly correlated variables (Pearson correlation > 0.6) were removed (e Table 2, e Figure 2). Among the remaining 41 variables, a total of 25 variables had missingness < 20% and were selected and scaled for computable phenotype determination. Log transformation was used for non-parametric variables.

Further, to impute the missing data in the 25 selected variables, multiple imputation with chained equation (MICE) was used (1). MICE is based on the Fully Conditional Specification (FCS) approach, where each incomplete variable is imputed by a separate model. Using FCS, MICE can impute mixes of continuous, binary, unordered categorical and ordered categorical data. In running MICE, we assumed that missing data is conditional on observed data and is following the pattern of “missing at random”.

1. **Consensus k-means clustering derivation of *nonsynonomous* cluster phenotypes**

Consensus k-means clustering was used to derive the phenotype membership of patients based on different number of clusters (k) as performed in the SENECA study. We then identified the optimal number of phenotypes (clusters) according to diagnostic plots.

To determine the membership of each patient, consensus clustering takes advantage of subsampling techniques so that perturbations of the original data can be simulated. In each subsampling run, the k-means algorithm was applied to the perturbed data sets for a given k. With this setting, the method calculates a “consensus value” for each pair of patients. This consensus value can be interpreted as the frequency the two patients are assigned to the same phenotype. It ranges from 0 to 1, with larger values indicating stronger consensus. Then, a hierarchical clustering is applied on consensus value matrix to obtain final cluster assignment for each subject.

To determine the optimal number of clusters, we assessed a combination of phenotype size, separation of the consensus matrix heatmaps, characteristics of the consensus cumulative distribution function plots, and adequate pairwise–consensus values between cluster members (eFigure 3 and 4). The consensus matrix heatmap is a plot having patients as both rows and columns. The consensus value is the frequency the two patients are assigned to the same phenotype among 1000 iterations. It ranges from 0 (white, interpreted as two patients are never clustered together) to 1 (dark blue interpreted as two patients are always clustered together). A clear separation of white and dark blue blocks in the heatmap is an indicator of good partitioning. The Consensus CDF plot shows the cumulative distribution functions of the consensus matrix for each k, estimated by a histogram of 100 bins. It is used to determine at what number of clusters the CDF reaches an approximate maximum; thus, consensus and cluster confidence is at a maximum at this k. It is usually used together with the Delta area plot to determine the optimal k. Usually, an “elbow” in the Delta area plot is an indicator of the optimal k. The Cluster-consensus plot shows the cluster-consensus value of clusters at each k. This is the mean of all pairwise consensus values between a cluster’s members. High values indicate a cluster has high stability and low values indicate a cluster has low stability. We used 0.5 as a cut off for diagnostic purposes.

Our choice of consensus k-means clustering was based on the following two reasons. Firstly, consensus clustering provides a solution to represent the most common assignment across multiple runs of a clustering algorithm. Therefore, it is able to provide qualitative and quantitative measurements for internal validation purposes, which contributes to determining the number and the stability of the discovered clusters. Secondly, we chose to use k-means as the inner-loop clustering algorithm based on the data structure indicated by the OPTICS plot (eFigure 3) (2). The OPTICS plot shows a smooth rise instead of clearly partitioned valleys, which implies that the data cannot be arranged into discrete natural groupings. In this case, a partitioning clustering method, such as k-means, is more appropriate than hierarchical clustering methods.

1. **Comparison of Latent class analysis derived groups to Consensus k means phenotypes**

To assess and confirm underlying derived phenotype structure and stability of individual assignment of the four phenotypes we applied a second method namely latent class analysis (LCA) (3) to the same dataset (eTable 4, eFigure 5-7) as was done in the SENECA study. After running LCA with different numbers of clusters, we separately used Bayesian information criteria (BIC), group size, membership of patients and clinical characteristics of phenotype groups (eTable 4) to confirm that four underlying phenotypes is optimal using LCA in our data (eFigure 6). We further visualize and find similar side by side Rank of variable contributions based on the LCA as with the consensus k means method (eFigure 5), and visualize reasonable patient membership overlap between consensus k means and LCA in an alluvial diagram (eFigure 7).

1. **Dissimilarity visualization of Consensus k means phenotypes**

In order to assess dissimilarity among the four derived consensus k means phenotypes we used 1) a t-distributed stochastic neighbor embedding (t-SNE) plot labeled by *a priori* outcomes of interest (Figure 2); 2) chord diagrams in terms of *a priori* clinical characteristics and organ dysfunction patterns (Figure 2); and 3) variable contributions to pairwise phenotype discrimination (eFigure 5).

1. **Heterogeneity of *a priori* biomarkers and primary outcomes among derived phenotypes**

Following the consensus k means phenotype determination, we correlated the derived phenotypes with their biomarkers (Figure 3, eTable 7 and 8, eFigure 10) and investigated their relationships to mortality (Table 2, Figure 4 and 5, eTable 9-11 eFigure 12), organ failure (Table 2, Figure 5, eFigure 11 and 13), and MOF pathobiology groups (Table 2, eFigure 14). To determine the correlation of the derived 24-hour phenotypes with 33 biomarkers of the host response (Figure 3, eTable 7, 8, 18 eFigure 10) we compared mean and standard deviation, median and interquartile range (IQR) for continuous data, and the ratio of the cases in binary data. The cytokine heatmap was used to present the log ratio of the median biomarker values for various markers of the host response. Red represents a greater median biomarker value for that phenotype compared with the median for the entire study cohort, whereas blue represents a lower median biomarker value compared with the median for the entire study cohort. Hierarchical clustering was used to identify similarities of cytokine patterns across the phenotypes. To investigate the relationship of the derived phenotypes to *a priori* primary outcomes (mortality, MOF groups), we estimated the association between derived phenotype and *a priori* outcomes with a multivariate model adjusting for demographic variables (age, sex, race, ethnicity) and PRISM score. We also generated phenotype specific curve plots to assess differences in mortality and number of organ failures over time (Figure 5, eFigure 13).

1. **Exploratory analysis of heterogeneity of treatment interactions with derived phenotypes**

Within each of the derived phenotypes, we first evaluated which of the 41 anti-inflammatory/ immunomodulatory and 3 organ support therapies given in the parent study by bedside clinicians were associated with survival in univariate logistic regression among the subset of patients who were given anti-inflammatory therapies by the bedside clinicians (eTable 12 and 13). For the 14 significant individual treatments (11 anti-inflammatory/immunomodulatory therapies and 3 organ support therapies) we further applied Elastic Net regression to investigate their interactive effects on survival within each of the derived phenotypes (Figure 6, eFig 15).^26^ Two treatment combinations in PedSep-D were found by elastic net regression analysis to have a interaction effect < 0.10 (Methylprednisolone and IVIG; and plasma exchange and ECMO) . We performed traditional multivariable logistic regression to determine 95% confidence intervals for interactions between methylprednisone and IVIG therapies in PedSep-D, combined Methylprednisolone + IVIG and PedSep-D membership, and plasma exchange and ECMO in PedSep-D (eTable 13-17).

1. **Other information**

For summary analysis, we presented continuous data as mean (SD) or median (IQR) and categorical data as count number (%). For comparison, we used Kruskal-Wallis tests for continuous data and the chi square test for categorical data. Fisher exact tests were applied for cells containing less than 5 samples. The threshold for statistical significance was less than 0.05 for two-sided tests after adjustment for multiple testing. Holm–Bonferroni correction was applied to correct for multiple testing. Analyses were performed with R version 3.6.2.

1. **Computable prediction tool of individual membership in phenotypes**

We also developed a computable tool ([www.pittsepsis.edu](http://www.pittsepsis.edu)) that allows one to categorize the phenotype of a new individual patient into one of the four derived phenotypes at the bedside. Specifically, we first used the same pipeline to standardize and normalize the 25 input variables of the new patient as described above. Then we calculated the Euclidean distance from this patient to the centroid of each phenotype derived from the unsupervised consensus k-means clustering. Comparing the distances to four phenotype centroids, we assign the patient to the phenotype with the shortest distance.

1. Newgard CD, Haukoos JS. Advanced statistics: missing data in clinical research—part 2: multiple imputation. Acad Emerg Med. 2007;14(7):669-678.

2. Ankerst M. OPTICS: ordering points to identify the clustering structure. SIGMOD Rec. 1999;28(2): 49-60. doi:10.1145/304181.304187.

3. Rindskopf D, Rindskopf W. The value of latent class analysis in medical diagnosis. Stat Med. 1986;5 (1):21-27. doi:10.1002/sim.4780050105.

**Supplementary Digital Content eTables 1-17 and eFigures 1-15**

eTable 1. List of all *a priori* candidate variables

eTable 2. Missing data (no., %) for demographic and day 1 clinical variables

eTable 3. Statistical comparison of demographic and day 1 clinical characteristics (n = 404)

eTable 4. Statistical output from latent class analysis

eTable 5. Recorded diagnoses according to four phenotypes

eTable 6. Statistical differences in recorded diagnoses counts among the four phenotypes

eTable 7. Biomarker levels at Day 1 in PedSep A, B, C, and D

eTable 8. Statistical comparison of biomarker levels measured at day 1 in PedSep-A, B, C, and D

eTable 9. Statistical Test Results of Subsequent Outcome Characteristics (n = 404)

eTable 10. Statistical test results of association between day 1 characteristics and mortality in PedSep-A to D

eTable 11. Statistical test results of association between biomarkers and mortality in PedSep A, B, C, and D

eTable 12. Statistical test result of 44 candidate therapies’ univariable association with outcome

eTable 13. Median duration of anti-inflammatory or immune medications used in at least five patients

eTable 14. Univariable adjusted mortality risks associated with Methylprednisolone and IVIG in PedSep-D

eTable 15. Interactions between Methylprednisone and IVIG treatments in PedSep-D

eTable 16 Univariable adjusted mortality risks in PedSep- B, C, and D with Methylprednisolone and IVIG

eTable 17 Interactions between PedSep D membership and combined IVIG + Methylprednisolone treatment

eFigure 1. CONSORT diagram in parent study (n = 404) used for machine learning analysis

eFigure 2. Heatmap of correlation between clinical variables for phenotyping

eFigure 3. OPTICS plots (N= 404)

eFigure 4. Consensus k clustering results

eFigure 5. Comparison of variables that contribute to clinical phenotypes using Consensus k-means and LCA

eFigure 6. Sensitivity analysis using latent class clustering (N=404), showing probabilities of phenotype assignment.

eFigure 7. Comparison of phenotype membership between Consensus k-means and LCA

eFigure 8. t-SNE plot of selected diagnoses according to phenotypes

eFigre 9. t-SNE plot of infection status according to phenotypes

eFigure 10. Comparison of selected inflammatory cytokines bar graphs across phenotypes

eFigure 11. Alluvial plot showing distribution of phenotypes across baseline OFI (N=404)

eFigure 12. Comparison of Cytokine Biomarkers That Contribute to Survival within Each Phenotype

eFigure 13. 28-day organ failure curve (0 for discharged survivor, 6 for discharged non-survivor)

eFigure 14. t-SNE plot of outcome, IPMOF, TAMOF, SMOF, and MAS (N = 404)

eFigure 15. Count of patients receiving 14 therapies alone and in combination, with outcome status

**eTable 1. List of all variables collected in parent study with reasons for exclusion from final 25 variable k means clustering modleing of nonsynonymous phenotypes**

| **Variable** | **Description of variable** | **Reason for exclusion** |
| --- | --- | --- |
| **Demographic** |  |  |
| Age |  |  |
| Sex |  |  |
| Race |  | Self-reported race with multiple overlapping groups |
| Ethnicity |  |  |
| Previous healthy |  |  |
| Surgery  Specific Diagnoses  Infection type or Infection site |  | High correlation with previously heathy status    Not reliably available at 24 hours |
| **PRISM^a^** |  |  |
| Low SBP | Lowest Systolic Blood Pressure |  |
| High Heart Rate | Highest Heart Rate |  |
| Low Temp | Lowest Temperature |  |
| High Temp | Highest Temperature |  |
| Pupillary Reflex | Number of pupils > 3 mm and fixed | Non-informative^b^ |
| GCS | The lowest GCS score |  |
| GCS Eye | GCS Eye | High correlation with GCS |
| GCS Verbal | GCS Verbal | High correlation with GCS |
| GCS Motor | GCS Motor | High correlation with GCS |
| Intubate | Intubated at GCS assessment |  |
| Low pH | Lowest pH | High missingness |
| Low PaO2 | Lowest PaO2 | High missingness |
| High PCO2 | Highest PCO2 | High missingness |
| Low Total CO2 | Lowest Total CO2 | High missingness |
| High Glucose | Highest Serum Glucose | High missingness |
| High Potassium | Highest Serum Potassium | High missingness |
| High Creatinine | Highest Creatinine | High correlation with Higher Creatinine |
| Low WBC | Lowest WBC | High missingness |
| Low Platelet | Lowest Platelets |  |
| High PT | Highest PT | High missingness |
| High PTT | Highest PTT | High missingness |
| High BUN | Highest Blood urea nitrogen | High missingness |
| **Labs** |  |  |
| High Creatinine | Creatinine | High correlation with Higher Creatinine |
| Higher Creatinine | Highest value from PRISM High Creatinine and High Creatinine |  |
| High PT | PT | High correlation with PT |
| Higher PT | Highest value from PRISM High PT and High PT | High missingness |
| Low Lymphocyte | Absolute lymphocyte count |  |
| Low Neutrophil | Absolute neutrophil count | High missingness |
| Low Hemoglobin | Hemoglobin |  |
| Low Platelet | Platelet count |  |
| High INR | International normalized ratio | High missingness |
| High Bilirubin | Total bilirubin | High missingness |
| High Lipase | Lipase | High missingness |
| High ALT | ALT | High missingness |
| High LDH | LDH | High missingness |
| High Triglycerides | Triglycerides | High missingness |
| **Organ failure** |  |  |
| OFI Cardio | Cardiovascular failure: Inotrope OR vasopressor infusion requirement. |  |
| OFI Pulm | Pulmonary failure: PaO2/FIO2 ratio of < 300 mm Hg AND mechanical ventilator requirement. |  |
| OFI Hepatic | Hepatic failure: ALT > 100 U/L AND either bilirubin > 1.0 mg/dL OR INR > 1.5 |  |
| OFI Renal | Renal failure: Creatine > 1mg/dL with Oliguria (urine output < 0.5 mL/kg/hr) |  |
| OFI Hemat | Hematologic failure: Platelet count < 100,000 cells/$\mu$L AND INR > 1.5 |  |
| OFI CNS | Central Nervous System failure: Glasgow coma score < 12 in absence of sedatives |  |
| OFI | Total number of organ failure |  |
| SIRS | Systemic Inflammatory Response Syndrome criteria |  |
| **Cytokine** |  |  |
| CRP |  |  |
| Ferritin |  |  |

a. PRISM: Pediatric Risk of Mortality Index. Variables record the worst physiologic values obtained in following 6 hour timeframe: 2 hours prior to ICU admission through 4 hour post ICU admission.

b. All samples had the same values.

**eTable 2. Missing data (no., %) for the 25 demographic and day 1 clinical variables used in the k means clustering modeling of nonsynonymous phenotypes**

| **Variable** | **No. of missing data (%)** |
| --- | --- |
| **Demographic** |  |
| Age | 0 (0) |
| Sex | 0 (0) |
| Ethnicity | 0 (0) |
| Previous healthy | 0 (0) |
| Surgery | 0 (0) |
| **Organ Dysfunction** |  |
| SIRS criteria | 0 (0) |
| OFI | 0 (0) |
| **Inflammation** |  |
| C-reactive protein | 4 (0.9) |
| Low Temperature | 2 (0.4) |
| High Temperature | 2 (0.4) |
| ALC | 79 (19.5) |
| Ferritin | 4 (0.9) |
| **Pulmonary** |  |
| Pulmonary OFI | 0 (0) |
| Intubation | 30 (7.4) |
| **Cardiovascular or Hemodynamic** |  |
| Heart rate | 0 (0) |
| Systolic blood pressure | 1 (0.2) |
| CV OFI | 0 (0) |
| **Renal** |  |
| Creatinine | 19 (4.7) |
| Renal OFI | 0 (0) |
| **Hepatic** |  |
| Hepatic OFI | 0 (0) |
| **Hematologic** |  |
| Hemoglobin | 48 (11.9) |
| Platelets | 36 (8.9) |
| Hematologic OFI | 0 (0) |
| **Other** |  |
| Glasgow Coma Scale score | 0 (0) |
| CNS OFI | 0 (0) |

Abbreviations: SIRS, systemic inflammatory response syndrome; OFI, organ failure index; ALC, absolute lymphocyte count; CNS, central nervous system

**eTable 3. Statistical test results of demographic and day 1 clinical characteristics among the phenotypes**

| **Characteristic^a^** | **Statistical test p-value** | | | | | | |
| --- | --- | --- | --- | --- | --- | --- | --- |
|  | **General** | **Pairwise** | | | | | |
|  |  | PedSep-A  vs  PedSep-B | PedSep-A    vs  PedSep-C | PedSep-A    vs  PedSep-D | PedSep-B  vs  PedSep-C | PedSep-B  vs  PedSep-D | PedSep-C  vs  PedSep-D |
| **Demographic** |  |  |  |  |  |  |  |
| Age | <0.001 | <0.001 | <0.001 | <0.001 | 0.023 | 0.917 | 0.206 |
| Sex | 0.014 | 0.017 | 0.934 | 0.392 | 0.364 | 0.964 | 0.964 |
| Ethnicity | 0.027 | 0.400 | 0.400 | 0.130 | 0.400 | 0.570 | 0.180 |
| Previous healthy | <0.001 | <0.001 | <0.001 | <0.001 | 1.000 | 1.000 | 1.000 |
| Surgery | <0.001 | 0.004 | 0.357 | 0.004 | 0.357 | 0.830 | 0.357 |
| **Organ Dysfunction** |  |  |  |  |  |  |  |
| SIRS criteria | 0.221 | 0.830 | 1.000 | 1.000 | 0.360 | 1.000 | 0.830 |
| OFI | <0.001 | <0.001 | 0.420 | <0.001 | <0.001 | <0.001 | <0.001 |
| **Inflammation** |  |  |  |  |  |  |  |
| C-reactive protein | <0.001 | <0.001 | <0.001 | 0.002 | 0.137 | 0.952 | 0.265 |
| Low Temperature | <0.001 | 0.002 | <0.001 | 0.108 | <0.001 | 0.298 | <0.001 |
| High Temperature | <0.001 | 0.080 | 0.002 | 0.594 | <0.001 | 0.389 | 0.012 |
| ALC | <0.001 | <0.001 | <0.001 | 0.001 | <0.001 | 0.782 | <0.001 |
| Ferritin | <0.001 | <0.001 | <0.001 | <0.001 | 0.001 | <0.001 | 0.187 |
| **Pulmonary** |  |  |  |  |  |  |  |
| Pulmonary OFI | <0.001 | 0.319 | <0.001 | 0.2584 | <0.001 | 0.053 | <0.001 |
| Intubation | <0.001 | <0.001 | <0.001 | 1.000 | <0.001 | <0.001 | <0.001 |
| **Cardiovascular or Hemodynamic** | | |  |  |  |  |  |
| Heart rate | <0.001 | <0.001 | <0.001 | 0.005 | 0.918 | 0.911 | 0.918 |
| Systolic blood pressure | <0.001 | <0.001 | 0.845 | 0.180 | <0.001 | 0.731 | 0.180 |
| CV OFI | <0.001 | <0.001 | <0.001 | <0.001 | 0.057 | 0.151 | 1.000 |
| **Renal** |  |  |  |  |  |  |  |
| Creatinine | <0.001 | <0.001 | <0.001 | <0.001 | 0.320 | <0.001 | <0.001 |
| Renal OFI | <0.001 | 1.000 | 1.000 | <0.001 | 1.000 | <0.001 | <0.001 |
| **Hepatic** |  |  |  |  |  |  |  |
| Hepatic OFI | <0.001 | 0.066 | 0.034 | <0.001 | 0.818 | 0.006 | 0.007 |
| **Hematologic** |  |  |  |  |  |  |  |
| Hemoglobin | 0.001 | 0.043 | 0.797 | 0.014 | 0.044 | 0.719 | 0.014 |
| Platelets | <0.001 | <0.001 | <0.001 | <0.001 | 0.005 | <0.001 | 0.004 |
| Hematologic OFI | <0.001 | 1.000 | 0.004 | <0.001 | 0.014 | <0.001 | <0.001 |
| **Other** |  |  |  |  |  |  |  |
| Glasgow Coma Scale score | <0.001 | <0.001 | <0.001 | 0.642 | <0.001 | 0.001 | <0.001 |
| CNS OFI | <0.001 | 0.016 | 0.891 | 0.092 | 0.002 | 0.918 | 0.017 |

Abbreviations: SIRS, systemic inflammatory response syndrome; OFI, organ failure index; ALC, absolute lymphocyte count; CNS, central nervous system ^a^Comparisons across all 4 phenotypes were performed using the Kruskal-Wallis test for continuous variables, the χ2 test for categorical variables, or the fisher’s exact test for cells with less than 5 patients.

**eTable 4. Statistical output from latent class analysis**

|  | **Statistic^a^** | | | | **Class size^b^ N, (%)** | | | | | |
| --- | --- | --- | --- | --- | --- | --- | --- | --- | --- | --- |
| **Class number** | AIC | BIC | Entropy^a^ | Median [IQR] (%) probability of membership | 1 | 2 | 3 | 4 | 5 | 6 |
| **2** | 44875 | 45199 | 0.917 | 100.0[99.9-100.0] | 308(76) | 96(24) | - | - | - | - |
| **3** | 44329 | 44817 | 0.872 | 99.8[97.1-100.0] | 212(52) | 129(32) | 63(16) | - | - | - |
| **4** | 43774 | 44426 | 0.904 | 99.8[97.4-100.0] | 144 (36) | 142(35) | 73(18) | 45(11) | - | - |
| **5** | 38542 | 39359 | 0.999 | 99.9[98.6-100.0] | 146(36) | 114(28) | 87(22) | 34(8) | 23(6) | - |
| **6** | 39540 | 40520 | 0.999 | 99.8[96.9-100.0] | 98(24) | 87(22) | 83(21) | 74(18) | 42(10) | 20(5) |

Abbreviations: AIC, Akaike information criterion; BIC, Bayesian information criteria; IQR, interquartile range

a AIC and BIC are information criteria for comparing models, where lower value suggests a better fit; Entropy is a measure between 0 and 1 measures success of classification, where a value closer to 1 implies a better fit.

b class size shows the number of samples assigned into each cluster, relatively large size of each cluster is preferred.

**eTable 5. Recorded diagnoses according to four phenotypes**

|  | **General** | **PedSep-A** | **PedSep-B** | **PedSep-C** | **PedSep-D** |
| --- | --- | --- | --- | --- | --- |
| Leukemia, N (%) | 31 (7.7) | 0 (0) | 4 (3.9)^a^ | 19 (17.3)^a,b^ | 8 (14.3)^a,b^ |
| Hemolytic Anemia, N (%) | 5 (1.2) | 0 (0) | 1 (1.0) | 2 (1.8) | 2 (3.6) |
| Rheumatic Disease, N (%) | 7 (1.7) | 0 (0) | 1 (1.0) | 4 (3.6)^a^ | 2 (3.6) |
| IBD, N (%) | 6 (1.5) | 0 (0) | 0 (0) | 3 (2.7) | 3 (5.4)^a,b^ |
| Renal Disease, N (%) | 6 (1.5) | 0 (0) | 3 (2.9) | 1 (0.9) | 2 (3.6) |
| Chromosome Abnormal, N (%) | 57 (14.1) | 15 (11.0) | 19 (18.6) | 10 (9.1) | 13 (23.2)^c^ |
| Metabolic Disease, N (%) | 15 (3.7) | 5 (3.7) | 8 (7.8) | 2 (1.8) | 0 (0) |
| Diabetes, N (%) | 4 (1.0) | 0 (0) | 2 (2.0) | 1 (0.9) | 1 (1.8) |
| Cardiovascular Disease, N (%) | 58 (14.4) | 18 (13.2) | 19 (18.6) | 11 (10.0) | 10 (17.9) |
| Cardiovascular Disease + Postoperative, N (%) | 19 (4.7) | 4 (2.9) | 8 (7.8) | 2 (1.8) | 5 (8.9) |
| Trauma, N (%) | 6 (1.5) | 3 (2.2) | 2 (2.0) | 1 (0.9) | 0 (0) |
| Short Gut, N (%) | 9 (2.2) | 0 (0) | 2 (2.0) | 6 (5.5)^a^ | 1 (1.8) |
| Liver Disease, N (%) | 13 (3.2) | 2 (1.4) | 2 (2.0) | 5 (4.5) | 4 (7.1) |
| Acute Bronchiolitis, N (%) | 11 (2.7) | 9 (6.6)^b,c^ | 1 (0.9) | 1 (0.9) | 0 (0) |

a The diagnosis this computable phenotype is significantly higher than PedSep-A (p-value < 0.05)

b The diagnosis characteristic of this computable phenotype is significantly higher than PedSep-B (p-value < 0.05)

c The diagnosis characteristic of this computable phenotype is significantly higher than PedSep-C (p-value < 0.05)

d The diagnosis characteristic of this computable phenotype is significantly higher than PedSep-D (p-value < 0.05)

Comparisons across all 4 computable phenotypes were performed using the χ2 test, or the fisher’s exact test (P < 0.05 for all comparisons after adjustment).

**eTable 6. Statistical differences in diagnoses counts among the four phenotypes**

|  | **General** | **PedSep-A vs PedSep-B** | **PedSep-A vs PedSep-C** | **PedSep-A vs PedSep-D** | **PedSep-B vs PedSep-C** | **PedSep-B vs PedSep-D** | **PedSep-C vs PedSep-D** |
| --- | --- | --- | --- | --- | --- | --- | --- |
| Leukemia | <0.001 | 0.033 | < 0.001 | < 0.001 | 0.002 | 0.027 | 0.665 |
| Hemolytic Anemia | 0.105 | 0.429 | 0.199 | 0.084 | 1.000 | 0.287 | 0.604 |
| Rheumatic Disease | 0.103 | 0.429 | 0.039 | 0.084 | 0.371 | 0.287 | 1.000 |
| IBD | 0.015 | 1.000 | 0.088 | 0.024 | 0.248 | 0.043 | 0.406 |
| Renal Disease | 0.142 | 0.077 | 0.447 | 0.084 | 0.353 | 1.000 | 0.263 |
| Chrom Abnormal | 0.031 | 0.141 | 0.773 | 0.051 | 0.069 | 0.632 | 0.024 |
| Metabolic Disease | 0.043 | 0.248 | 0.465 | 0.324 | 0.052 | 0.051 | 0.550 |
| Diabetes | 0.300 | 0.183 | 0.447 | 0.292 | 0.609 | 1.000 | 1.000 |
| Cardiovascular Disease | 0.272 | 0.339 | 0.560 | 0.549 | 0.109 | 1.000 | 0.233 |
| Cardiovascular + Postoperative | 0.048 | 0.132 | 0.694 | 0.125 | 0.052 | 0.773 | 0.044 |
| Trauma | 0.629 | 1.000 | 0.630 | 0.557 | 0.609 | 0.540 | 1.000 |
| Short Gut | 0.038 | 0.183 | 0.007 | 0.292 | 0.283 | 1.000 | 0.425 |
| Liver Disease | 0.155 | 1.000 | 0.248 | 0.061 | 0.448 | 0.187 | 0.488 |
| Acute Bronchiolitis | 0.003 | 0.011 | 0.026 | 0.061 | 1.000 | 1.000 | 1.000 |

Comparisons across all 4 phenotypes were performed using the χ2 test for categorical variables, or the fisher’s exact test for cells with less than 5 patients.

**eTable 7. Biomarkers measured at day 1 by phenotype**

| **Biomarker^a^** | **Total** | **Phenotype** | | | |
| --- | --- | --- | --- | --- | --- |
|  |  | **PedSep-A** | **PedSep-B** | **PedSep-C** | **PedSep-D** |
| ADAMTS13, % | 71.0 (56.0, 88.0) | 82.5 (65.0, 95.0) | 71.0 (57.0, 89.3) | 69.0 (52.5, 84.5) | 54.0 (38.0, 66.5) |
| SFasLg, pg/ml | 44.9  (29.0, 73.2) | 58.4  (37.2, 84.6) | 43.2  (31.1, 78.1) | 38.0  (25.0, 65.9) | 36.7  (20.9, 49.3) |
| Ex vivo TNF-α,  pg/ml | 427.8  (97.0, 1023.3) | 689.7  (347.1, 1049.2) | 331.1  (99.2, 806.8) | 212.3  (35.7, 668.0) | 278.0  (53.0, 1049.2) |
| TNF-α, pg/ml | 74.9  (56.2, 105.7) | 69.0  (51.9, 85.1) | 74.9  (55.4, 101.8) | 76.2  (55.4, 108.6) | 102.2  (81.4, 131.7) |
| sCD163, pg/ml | 294096  (195700, 496348) | 223123  (163123, 323365) | 309800  (185699, 508775) | 345238  (248348, 572766) | 668162  (280407, 897784) |
| IFN-β, pg/ml | 6.4 (6.4, 8.2) | 6.4 (6.4, 7.2) | 6.4 (6.4, 9.9) | 6.4 (6.4, 10.8) | 6.4 (6.4, 6.4) |
| IL-22, pg/ml | 26.0 (20.1, 34.2) | 22.4 (17.8, 29.5) | 28.0 (21.3, 36.9) | 27.1 (20.1, 34.2) | 31.9 (24.8, 49.2) |
| IL-18, pg/ml | 424.5 (255.2, 732.9) | 326.4 (217.2, 480.9) | 461.1 (279.4, 792.1) | 576.4 (300.2, 1100.5) | 518.4 (344.6, 857.1) |
| IL-18BP, pg/ml | 16083.6 (9107.0, 29173.7) | 9649.8 (6141.4, 16025.1) | 16084 (9513, 26918) | 22654 (13694, 34513) | 30713 (18114, 40878) |
| MIG/CXCL9, pg/ml | 801.2 (428.4, 2013.6) | 576.2 (378.0, 1008.5) | 809.8 (432.4, 1963.7) | 1125.3 (496.6, 2470.9) | 2047.0 (649.8, 4354.5) |
| IL-1β, pg/ml | 2.8 (2.4, 3.3) | 2.6 (2.1, 3.2) | 2.8 (2.3, 3.2) | 2.9 (2.4, 3.3) | 2.9 (2.5, 3.3) |
| IL-4, pg/ml | 4.7 (3.5, 6.5) | 4.9 (3.5, 6.3) | 4.9 (3.9, 6.8) | 4.7 (3.5, 6.7) | 4.3 (3.5, 6.4) |
| IL-6, pg/ml | 8.8  (6.5, 19.0) | 6.9  (5.8, 10.0) | 8.7  (6.5, 25.2) | 11.1  (7.0, 27.2) | 16.8  (8.4, 43.8) |
| IL-8, pg/ml | 55.0  (31.4, 108.6) | 41.2  (27.7, 66.7) | 57.1  (34.7, 113.7) | 57.5  (35.1, 127.9) | 123.5  (71.2, 468.6) |
| IL-10, pg/ml | 22.5 (17.5, 33.4) | 19.3 (15.4, 24.6) | 22.5 (18.6, 37.1) | 23.7 (18.1, 37.5) | 32.8 (24.6, 73.3) |
| IL-13, pg/ml | 3.1 (3.1, 3.9) | 3.1 (3.1, 4.2) | 3.1 (3.1, 3.4) | 3.1 (3.1, 4.3) | 3.1 (3.1, 3.4) |
| IL-17A, pg/ml | 19.1 (16.5, 23.4) | 17.4 (15.6, 21.7) | 19.1 (16.5, 26.0) | 20.9 (16.7, 26.8) | 19.1 (16.5, 23.4) |
| IFN-γ, pg/ml | 2.8 (2.8, 2.8) | 2.8 (2.8, 3.0) | 2.8 (2.8, 3.0) | 2.8 (2.8, 3.0) | 2.8 (2.8, 3.2) |
| IP-10/CXCL10,  pg/ml | 727.8  (343.9, 1963.7) | 494.0  (287.3, 1725.7) | 705.8  (259.7, 1585.7) | 967.7  (409.0, 2334.1) | 789.5  (466.8, 2109.7) |
| MCP-1/CCL2, pg/ml | 142.5  (70.9, 367.6) | 103.6  (49.7, 197.2) | 169.8  (83.9, 383.3) | 184.4  (88.7, 474.6) | 240.7  (114.5, 1623.0) |
| MIP-1α, pg/ml | 0.6 (0.6, 7.7) | 0.6 (0.6, 0.6) | 0.6 (0.6, 8.1) | 0.6 (0.6, 9.0) | 6.6 (2.8, 16.1) |
| MIP-1β, pg/ml | 45.9 (31.4, 70.4) | 42.4 (28.1, 56.3) | 45.7 (31.9, 73.9) | 44.6 (32.9, 77.8) | 58.4 (47.9, 95.0) |
| MCP-3, pg/ml | 92.4 (92.4, 166.0) | 92.4 (92.4, 147.8) | 92.4 (92.4, 166.0) | 119.5 (92.4, 166.0) | 119.5 (92.4, 180.6) |
| IFN-α2, pg/ml | 125.7 (105.8, 140.2) | 124.3 (105.8, 140.2) | 125.7 (108.8, 144.4) | 125.7 (105.8, 140.2) | 120.0 (105.8, 137.9) |
| IL-1α, pg/ml | 9.4 (9.4, 13.2) | 9.4 (9.4, 9.9) | 9.4 (9.4, 16.4) | 9.4 (9.4, 15.6) | 9.4 (9.4, 13.2) |
| IL-2RA, pg/ml | 378.8 (243.0, 623.2) | 345.2 (237.6, 511.6) | 385.3 (206.6, 683.1) | 380.6 (243.1, 731.5) | 449.7 (307.9, 747.5) |
| IL-3, pg/ml | 612.2 (529.0, 724.4) | 624.4 (496.1, 734.6) | 636.6 (529.0, 724.4) | 612.2 (529.0, 724.4) | 586.4 (496.1, 693.0) |
| IL-16, pg/ml | 569.8 (410.2, 763.0) | 544.4 (391.2, 677.2) | 590.4 (435.2, 770.2) | 529.4 (382.7, 704.3) | 858.0 (592.6, 1246.4) |
| M-CSF, pg/ml | 30.0 (17.0, 55.3) | 20.7 (14.2, 33.6) | 30.6 (20.3, 54.6) | 34.4 (20.3, 58.9) | 79.9 (46.2, 122.7) |
| SCF, pg/ml | 160.2 (118.8, 244.4) | 141.6 (115.0, 199.9) | 158.2 (113.8, 226.8) | 151.5 (114.2, 238.8) | 326.1 (227.5, 504.1) |
| TRAIL, pg/ml | 36.6 (27.9, 54.2) | 42.9 (32.9, 65.5) | 35.4 (25.4, 54.5) | 35.4 (29.1, 45.4) | 27.9 (24.1, 40.4) |
| CRPH, mg/dL | 9.8 (3.3, 17.1) | 4.3 (1.2, 12.4) | 10.1 (4.8, 19.3) | 14.3 (7.5, 21.7) | 10.7 (3.4, 20.7) |
| Ferritin, ng/mL | 218.0 (98.0, 625.3) | 125.4 (69.8, 207.8) | 223.1 (116.5, 544.2) | 405.5 (176.2, 1485.7) | 610.0 (221.1, 2482.0) |

^a^ All biomarkers are measured one time concomitantly in the first day. Values in table are summarized as median (IQR)

**eTable 8. Statistical test of differences of biomarkers among phenotypes measured at Day 1**

| **Biomarker^a^** | **General** | **Pairwise** | | | | | |
| --- | --- | --- | --- | --- | --- | --- | --- |
|  |  | **PedSep-A**  **vs**  **PedSep-B** | **PedSep-A**  **vs**  **PedSep-C** | **PedSep-A**  **vs**  **PedSep-D** | **PedSep-B**  **vs**  **PedSep-C** | **PedSep-B**  **vs**  **PedSep-D** | **PedSep-C**  **vs**  **PedSep-D** |
| ADAMTS13 | <0.001 | 0.033 | 0.001 | <0.001 | 1.000 | <0.001 | <0.001 |
| sFasLg | <0.001 | 0.140 | <0.001 | <0.001 | 0.419 | 0.111 | 1.000 |
| Ex vivo TNF-α | <0.001 | 0.002 | <0.001 | 0.061 | 0.506 | 1.000 | 1.000 |
| TNF-α | <0.001 | 0.321 | 0.152 | <0.001 | 1.000 | <0.001 | 0.002 |
| sCD163 | <0.001 | 0.008 | <0.001 | <0.001 | 0.680 | 0.0015 | 0.021 |
| IFN-β | 0.068 | 0.400 | 0.320 | 1.000 | 1.000 | 0.400 | 0.430 |
| IL-22 | <0.001 | 0.005 | 0.004 | <0.001 | 1.000 | 0.270 | 0.139 |
| IL-18 | <0.001 | 0.005 | <0.001 | <0.001 | 0.695 | 1.000 | 1.000 |
| IL-18BP | <0.001 | <0.001 | <0.001 | <0.001 | 0.016 | <0.001 | 0.227 |
| MIG/CXCL9 | <0.001 | 0.053 | <0.001 | <0.001 | 1.000 | 0.025 | 0.207 |
| IL-1β | 0.071 | 1.000 | 0.130 | 0.250 | 1.000 | 1.000 | 1.000 |
| IL-4 | 0.590 | 1.000 | 1.000 | 1.000 | 1.000 | 1.000 | 1.000 |
| IL-6 | <0.001 | 0.020 | <0.001 | <0.001 | 0.759 | 0.023 | 0.258 |
| IL-8 | <0.001 | 0.008 | 0.002 | <0.001 | 1.000 | <0.001 | <0.001 |
| IL-10 | <0.001 | 0.003 | <0.001 | <0.001 | 1.000 | 0.003 | 0.012 |
| IL-13 | 0.824 | 1.000 | 1.000 | 1.000 | 1.000 | 1.000 | 1.000 |
| IL-17A | <0.001 | 0.010 | <0.001 | 0.862 | 1.000 | 1.000 | 0.577 |
| IFN-γ | 0.998 | 1.000 | 1.000 | 1.000 | 1.000 | 1.000 | 1.000 |
| IP-10/CXCL10 | 0.013 | 1.000 | 0.024 | 0.133 | 0.267 | 0.817 | 1.000 |
| MCP-1/CCL2 | <0.001 | 0.003 | <0.001 | <0.001 | 1.000 | 0.079 | 0.371 |
| MIP-1α | <0.001 | <0.001 | <0.001 | <0.001 | 1.000 | 0.006 | 0.007 |
| MIP-1β | <0.001 | 0.168 | 0.093 | <0.001 | 1.000 | 0.037 | 0.084 |
| MCP-3 | 0.309 | 1.000 | 0.930 | 0.660 | 1.000 | 1.000 | 1.000 |
| IFN-α2 | 0.803 | 1.000 | 1.000 | 1.000 | 1.000 | 1.000 | 1.000 |
| IL-1α | 0.500 | 1.000 | 1.000 | 1.000 | 1.000 | 1.000 | 1.000 |
| IL-2RA | 0.021 | 1.000 | 0.462 | 0.007 | 1.000 | 0.565 | 1.000 |
| IL-3 | 0.596 | 1.000 | 1.000 | 1.000 | 1.000 | 1.000 | 1.000 |
| IL-16 | <0.001 | 0.318 | 1.000 | <0.001 | 0.488 | <0.001 | <0.001 |
| M-CSF | <0.001 | <0.001 | <0.001 | <0.001 | 1.000 | <0.001 | <0.001 |
| SCF | <0.001 | 0.760 | 0.850 | <0.001 | 1.000 | <0.001 | <0.001 |
| TRAIL | <0.001 | 0.032 | 0.003 | <0.001 | 1.000 | 0.104 | 0.047 |
| CRPH | <0.001 | <0.001 | <0.001 | 0.002 | 0.137 | 0.952 | 0.265 |
| Ferritin | <0.001 | <0.001 | <0.001 | <0.001 | <0.001 | <0.001 | 0.187 |

All biomarkers are measured in the first day.

**eTable 9. Statistical test results of differences in subsequent outcome characteristics among the phenotypes**

|  | **Statistical test p-value** | | | | | | | | | | |  |
| --- | --- | --- | --- | --- | --- | --- | --- | --- | --- | --- | --- | --- |
| **Characteristic^a^** | **General** | | **Pairwise** | | | | | | | | |  |
|  |  | **PedSep-A**  **vs**  **PedSep-B** | | **PedSep-A**  **vs**  **PedSep-C** | | **PedSep-A**  **vs**  **PedSep-D** | | **PedSep-B vs**  **PedSep-C** | | **PedSep-B**  **vs**  **PedSep-D** | **PedSep-C**  **vs**  **PedSep-D** | |
| **MOF Empirical Phenotypes** | | | | | | | | | | | |  |
| SMOF | <0.001 | | 1.000 | | 1.000 | | 0.003 | | 1.000 | 0.008 | 0.027 |  |
| TAMOF | <0.001 | | 0.017 | | 0.173 | | <0.001 | | 0.321 | <0.001 | <0.001 |  |
| IPMOF | <0.001 | | 0.00083 | | 0.064 | | <0.001 | | 0.446 | 0.446 | 0.064 |  |
| MAS | <0.001 | | 0.240 | | 0.390 | | <0.001 | | 0.680 | <0.001 | <0.001 |  |
| NPMOF | <0.001 | | 1.000 | | 0.489 | | <0.001 | | 1.000 | <0.001 | 0.003 |  |
| **Infections** | | | | | | | | | | | |  |
| Bacterial infection | 0.440 | | 1.000 | | 1.000 | | 1.000 | | 1.000 | 1.000 | 1.000 |  |
| Viral infection | <0.001 | | 0.002 | | 0.002 | | 0.002 | | 1.000 | 1.000 | 1.000 |  |
| Fungal infection | 0.003 | | 1.000 | | 1.000 | | 0.140 | | 1.000 | 0.510 | 0.190 |  |
| Culture negative | 0.049 | | 0.096 | | 0.432 | | 0.336 | | 1.000 | 1.000 | 1.000 |  |
| **Sites** | | | | | | | | | | | |  |
| Blood | <0.001 | | 1.000 | | 0.023 | | 0.023 | | 0.023 | 0.019 | 1.000 |  |
| Lung | 0.006 | | 0.634 | | 0.245 | | 0.634 | | 0.014 | 0.185 | 0.963 |  |
| Urine | 0.644 | | 1.000 | | 1.000 | | 1.000 | | 1.000 | 1.000 | 1.000 |  |
| **Organ Support** | | | | | | | | | | | |  |
| MechVent | <0.001 | | 1.000 | | <0.001 | | 0.302 | | <0.001 | 0.302 | 0.013 |  |
| ECMO | 0.006 | | 0.629 | | 0.983 | | 0.015 | | 0.983 | 0.629 | 0.112 |  |
| CRRT | <0.001 | | 0.067 | | 0.067 | | <0.001 | | 1.000 | <0.001 | <0.001 |  |
| **Anti-inflammatory therapies** | | | | | | | | | | | |  |
| Dexamethasone | <0.001 | | 0.057 | | <0.001 | | 0.002 | | 0.561 | 0.561 | 0.926 |  |
| Methylprednisolone | 0.009 | | 0.034 | | 0.086 | | 0.203 | | 1.000 | 1.000 | 1.000 |  |
| IVIG | <0.001 | | 0.300 | | 0.002 | | <0.001 | | 0.231 | 0.054 | 0.496 |  |
| Methylprednisolone + IVIG | 0.019 | | 0.460 | | 0.029 | | 0.012 | | 0.154 | 0.105 | 0.787 |  |
| Plasma exchange | <0.001 | | 1.000 | | 1.000 | | 0.002 | | 1.000 | 0.006 | 0.002 |  |
| ECMO + Plasma exchange | 0.207 | | 1.000 | | 1.000 | | 0.094 | | 1.000 | 0.298 | 0.299 |  |
| **Outcome** | | | | | | | | | | | |  |
| Length of Stay | <0.001 | | 0.365 | | 0.010 | | 0.052 | | 0.003 | 0.259 | <0.001 |  |
| Mortality | <0.001 | | 0.015 | | 0.023 | | <0.001 | | 0.826 | 0.006 | 0.002 |  |
| PICU-free Days | <0.001 | | 0.329 | | 0.038 | | <0.001 | | 0.012 | 0.003 | <0.001 |  |

Abbreviations: SMOF, sequential liver failure associated multiple organ failure; TAMOF, thrombocytopenia associated multiple organ failure; IPMOF, immunoparalysis associated multiple organ failure; MAS, macrophage activation syndrome; NPMOF, new or progressive multiple organ failure; IQR, interquartile range; MechVent, Mechanical Ventilation; ECMO, Extracorporeal Membrane Oxygenation; CRRT, Continuous Renal Replacement Therapies; IVIG, intravenous gamma globulin

^a^ Comparisons across all 4 phenotypes were performed using the Kruskal-Wallis test for continuous variables, the χ2 test for categorical variables, or the fisher’s exact test for cells with less than 5 patient

**eTable 10. Statistical test result of association between day 1 characteristics and mortality**

|  | **Phenotype^c^** | | | |
| --- | --- | --- | --- | --- |
| **Characteristic** | **PedSep-A** | **PedSep-B** | **PedSep-C** | **PedSep-D** |
| **Demographic** |  |  |  |  |
| Age | 0.45 | 0.39 | 0.55 | 0.78 |
| Sex | 1.00 | 1.00 | 0.80 | 0.26 |
| Ethnicity | 0.58 | 0.67 | 0.55 | 0.61 |
| Previous healthy | 1.00 | 0.58 | 0.89 | 0.02^b^ |
| Surgery | 1.00 | 0.56 | 1.00 | 0.77 |
| **Organ Dysfunction** |  |  |  |  |
| SIRS criteria | 0.86 | 0.85 | 0.74 | 0.20 |
| OFI | 0.75 | 0.67 | 0.23 | 0.20 |
| **Inflammation** |  |  |  |  |
| C-reactive protein | 0.34 | 0.13 | 0.52 | 0.68 |
| Low Temperature | 0.45 | 0.44 | 0.61 | 0.30 |
| High Temperature | 0.71 | 0.4 | 0.02^b^ | 0.80 |
| ALC | 0.89 | 0.35 | 0.02^b^ | 0.23 |
| Ferritin | 0.64 | 0.95 | 0.03^a^ | <0.01^a^ |
| **Pulmonary** |  |  |  |  |
| Pulmonary OFI | 1.00 | 1.00 | 0.06 | 0.33 |
| Intubation | 0.92 | 0.61 | 1.00 | 0.86 |
| **Cardiovascular or Hemodynamic** |  |  |  |  |
| Heart rate | 0.57 | 0.53 | 0.07 | 0.95 |
| Systolic blood pressure | 0.96 | 1.00 | <0.01^a^ | 0.08 |
| Cardiovascular OFI | 1.00 | 0.74 | 0.45 | 0.08 |
| **Renal** |  |  |  |  |
| Creatinine | 0.08 | 0.14 | 0.44 | 0.86 |
| Renal OFI | - | - | - | 0.34 |
| **Hepatic** |  |  |  |  |
| Hepatic OFI | 1.00 | 0.54 | 0.67 | 0.87 |
| **Hematologic** |  |  |  |  |
| Hemoglobin | 0.55 | 0.33 | 0.47 | 0.91 |
| Platelets | 0.34 | 0.89 | 0.16 | 0.26 |
| Hematologic OFI | - | - | 1.00 | 0.58 |
| **Other** |  |  |  |  |
| Glasgow Coma Scale score | 0.32 | 0.89 | 0.39 | 0.30 |
| CNS OFI | 0.01^a^ | 0.62 | 0.89 | 1.00 |

Abbreviations: SIRS, systemic inflammatory response syndrome; OFI, organ failure index; ALC, absolute lymphocyte count; CNS, central nervous system

^a^ Non-survivors have significantly higher value (proportion) of characteristic than survivors.

^b^ Non-survivors have significantly lower value (proportion) of characteristic than survivors.

^c^ Kruskal-Wallis or chi-square p-value, as appropriate, comparing non-survivors and survivors.

Cells without p-value result from 0 counts of individuals in tested group.

**eTable 11. Statistical test results of association between biomarkers and mortality**

| **Biomarker** | **Phenotype^c^** | | | |
| --- | --- | --- | --- | --- |
|  | **PedSep-A** | **PedSep-B** | **PedSep-C** | **PedSep-D** |
| CRPH | 0.34 | 0.13 | 0.52 | 0.68 |
| Ferritin | 0.64 | 0.95 | 0.03^a^ | <0.01^a^ |
| ADAMTS13 | 0.25 | 0.43 | 0.13 | 0.84 |
| sFasLg | 0.35 | 0.36 | 0.76 | 0.04^b^ |
| Ex vivo TNF-α | 0.31 | 0.71 | 0.88 | 1.00 |
| TNF-α | 0.04^b^ | 0.68 | 0.80 | 0.74 |
| sCD163 | 0.87 | 0.52 | 0.08 | 0.14 |
| IFN-β | 0.32 | 0.17 | 0.54 | 0.34 |
| IL-22 | 0.96 | 0.61 | 0.76 | 0.58 |
| IL-18 | 0.07 | 0.29 | 0.96 | 0.23 |
| IL-18BP | 0.09 | 0.06 | 0.62 | 0.06 |
| MIG/CXCL9 | 0.25 | 0.17 | 0.67 | 0.35 |
| IL-1β | 0.51 | 0.30 | 0.61 | 0.82 |
| IL-4 | 0.71 | 0.10 | 0.75 | 0.96 |
| IL-6 | 0.16 | 0.04^a^ | 0.26 | 0.99 |
| IL-8 | 0.16 | 0.02^a^ | <0.01^a^ | 0.01^a^ |
| IL-10 | 0.33 | 0.75 | 0.11 | <0.01^a^ |
| IL-13 | 0.88 | 0.40 | 0.58 | 0.66 |
| IL-17A | 0.12 | 0.88 | 0.44 | 0.71 |
| IFN-γ | 0.25 | 0.06 | 0.38 | 0.15 |
| IP-10/CXCL10 | 0.29 | 0.08 | 0.07 | 0.21 |
| MCP-1/CCL2 | 0.36 | <0.01^a^ | 0.06 | 0.11 |
| MIP-1α | 0.36 | 0.07 | 0.14 | <0.01^a^ |
| MIP-1β | 0.12 | 0.06 | 0.38 | 0.10 |
| MCP-3 | 0.03^a^ | 0.90 | 0.07 | 0.10 |
| IFN-α2 | 0.19 | 0.81 | 0.65 | 0.46 |
| IL-1α | 0.18 | 0.20 | 0.54 | 0.68 |
| IL-2RA | <0.01^b^ | 0.64 | 0.81 | 0.71 |
| IL-3 | 0.73 | 0.19 | 0.82 | 0.92 |
| IL-16 | 0.56 | 0.63 | 0.12 | 0.44 |
| M-CSF | 0.22 | 0.17 | 0.18 | 0.61 |
| SCF | 0.17 | 0.73 | 0.16 | 0.10 |
| TRAIL | 0.72 | 0.73 | 0.98 | 0.61 |

^a^ Non-survivors have significantly higher value of biomarker than survivors.

^b^ Non-survivors have significantly lower value of biomarker than survivors.

^c^ Kruskal-Wallis p-value comparing non-survivors and survivors.

**eTable 12. Univariable association of 44 therapies with mortality in the subset of patients given anti-inflammatory therapies**

| Therapy | General | PedSep-A | PedSep-B | PedSep-C | PedSep-D |
| --- | --- | --- | --- | --- | --- |
| ANAKINRA, p-value (No.) | 0.017 (5) | - (0) | 1 (1) | 0.055 (3) | 0.367 (1) |
| BECLOMETHASONE, p-value (No.) | 1 (2) | - (0) | - (0) | 1 (2) | - (0) |
| BORTEZOMIB, p-value (No.) | 1 (1) | - (0) | - (0) | - (0) | 1 (1) |
| CAMPATH, p-value (No.) | 1 (2) | - (0) | 1 (1) | 1 (1) | - (0) |
| CARBOPLATIN, p-value (No.) | 1 (1) | 1 (1) | - (0) | - (0) | - (0) |
| CELLCEPT, p-value (No.) | 1 (3) | - (0) | - (0) | 1 (1) | 0.526 (2) |
| CISPLATIN, p-value (No.) | 1 (1) | - (0) | - (0) | 1 (1) | - (0) |
| CYCLOPHOSPHAMIDE, p-value (No.) | 1 (1) | - (0) | - (0) | 1 (1) | - (0) |
| CYCLOSPORINE, p-value (No.) | 0.017 (2) | - (0) | - (0) | 0.147 (1) | 0.367 (1) |
| CYTARABINE, p-value (No.) | 0.342 (3) | - (0) | - (0) | 0.274 (2) | 1 (1) |
| CYTOGAM, p-value (No.) | 0.130 (1) | - (0) | - (0) | - (0) | 0.367 (1) |
| DAUNORUBICIN, p-value (No.) | 1 (1) | - (0) | - (0) | - (0) | 1 (1) |
| DEXAMETHASON, p-value (No.) | 0.580 (94) | 1 (50) | 1 (22) | 0.004 (14) | 1 (8) |
| DOXORUBICIN, p-value (No.) | 1 (1) | - (0) | - (0) | 1 (1) | - (0) |
| ENBREL, p-value (No.) | 0.130 (1) | - (0) | - (0) | 0.147 (1) | - (0) |
| EPOETIN ALFA, p-value (No.) | 1 (1) | - (0) | - (0) | - (0) | 1 (1) |
| ETANERCEPT, p-value (No.) | 1 (1) | 1 (1) | - (0) | - (0) | - (0) |
| ETOPOSIDE, p-value (No.) | 0.128 (5) | 1 (1) | - (0) | 0.020 (2) | 0.526 (2) |
| FLUDROCORTISONE, p-value (No.) | 1 (1) | 1 (1) | - (0) | - (0) | - (0) |
| FLUOROURACIL, p-value (No.) | 1 (1) | - (0) | - (0) | 1 (1) | - (0) |
| HYDROCORTISONE, p-value (No.) | 0.061 (172) | 1 (36) | 1 (48) | 1 (50) | 0.724 (38) |
| HYDROXYCHLOROQUINE, p-value (No.) | 1 (2) | 1 (1) | - (0) | 1 (1) | - (0) |
| HYDROXYUREA, p-value (No.) | 0.017 (2) | - (0) | - (0) | - (0) | 0.130 (2) |
| IMMUNOGLOBULIN G, p-value (No.) | 0.001 (51) | 1 (6) | 0.113 (10) | 0.025 (19) | 0.537 (16) |
| INFLIXIMAB, p-value (No.) | 0.130 (1) | - (0) | - (0) | 0.147 (1) | - (0) |
| METHYLPREDNISOLONE, p-value (No.) | 0.862 (117) | 1 (54) | 0.714 (23) | 0.004 (24) | 0.754 (16) |
| MYCOPHENOLATE, p-value (No.) | 0.001 (8) | - (0) | 1 (1) | 0.009 (4) | 0.546 (3) |
| NEUPOGEN, p-value (No.) | 0.001 (23) | - (0) | 0.241 (2) | 0.012 (12) | 0.708 (9) |
| PREDNISOLONE, p-value (No.) | 0.781 (32) | 1 (14) | 1 (5) | 0.612 (9) | 1 (4) |
| PROGRAF, p-value (No.) | 0.243 (2) | - (0) | - (0) | 0.274 (2) | - (0) |
| PULMICORT, p-value (No.) | 1 (3) | 1 (1) | 1 (1) | 1 (1) | - (0) |
| RASBURICASE, p-value (No.) | 1 (1) | - (0) | - (0) | - (0) | 1 (1) |
| RITUXIMAB, p-value (No.) | 1 (2) | 1 (1) | 1 (1) | - (0) | - (0) |
| SARGRAMOSTIM, p-value (No.) | 0.130 (1) | - (0) | - (0) | - (0) | 0.367 (1) |
| SIROLIMUS, p-value (No.) | 1 (2) | - (0) | - (0) | 1 (1) | 1 (1) |
| SYMBICORT, p-value (No.) | 1 (1) | 1 (1) | - (0) | - (0) | - (0) |
| TACROLIMUS, p-value (No.) | 0.042 (16) | 1 (1) | 1 (3) | 0.021 (5) | 1 (7) |
| THYMOGLOBULIN, p-value (No.) | 0.130 (1) | - (0) | - (0) | 0.147 (1) | - (0) |
| TOCILIZUMAB, p-value (No.) | 1 (1) | - (0) | - (0) | 1 (1) | - (0) |
| VINCRISTINE, p-value (No.) | 1 (1) | - (0) | - (0) | 1 (1) | - (0) |
| Plasma exchange, p-value (No.) | 0.047 (25) | 1 (5) | 0.067 (4) | 1 (4) | 1 (12) |
| MechVent, p-value (No.) | 0.10 (366) | 1 (134) | 1 (101) | 0.032 (79) | 1 (52) |
| ECMO, p-value (No.) | 0.001 (30) | 0.11 (5) | 0.001 (9) | 0.110 (6) | 0.073 (10) |
| CRRT, p-value (No.) | 0.001 (52) | 1 (1) | 0.003 (7) | 0.14 (7) | 0.23 (37) |

ues of 2-6 columns of table present p values from statistical tests and number of patients treated by each therapy in each

phenotype. A p value less than 0.05 indicates a significant association between individual therapy and mortality. “-” indicates no patient from a specific phenotype treated by this therapy. Therapies are selected for combination effect analysis if a significant association is detected in patients from at least one of four phenotypes or general population.

| **eTable 13 Anti-inflammatory/Immune Medications Duration median [IQR] administered to at least five patients identified in eTable 12.** | | | |
| --- | --- | --- | --- |
|  | **Frequency of Use on Study n/N (%)** |  | **Duration** |
| Any Immune Medication | 306/404 (76%) |  | -- |
|  |  |  |  |
| Methylprednisolone | 117/306 (38%) |  | 5 [2, 7] |
| Dexamethasone | 94/306 (30%) |  | 1 [1, 3] |
| Immunoglobulin G | 51/306 (17%) |  | 1 [0, 2] |
| G-CSF (Granulocyte colony stimulating factor) | 23/306 (8%) |  | 7 [4, 15] |
| Tacrolimus | 18/306 (6%) |  | 9.5 [3, 18] |
| Mycophenolate | 8/306 (3%) |  | 4 [2, 7] |
| Anakinra | 5/306 (2%) |  | 11 [10, 16] |
| Etoposide | 5/306 (2%) |  | 6 [3, 17] |
|  |  |  |  |
| Medication administrations were recorded on study Days 0-28 inclusive. Medication start and stop dates were truncated Day 0 and Day 28 or discharge, whichever came first, respectively. Duration is defined as the sum of calendar days on study the patient was receiving immune medications. Each medication duration summary only includes patients receiving the medication. Median [Q1, Q3] are reported. | | | |

**eTable 14 Adjusted univariate logistic regression models comparing the effects of Methylprednisolone to no Methylprednisolone, IVIG to no IVIG, and Methylprednisolone + IVIG together to no combination therapy in PedSep-D**

|  | PedSep-D | | |
| --- | --- | --- | --- |
|  | Mortality rate (N) | Odds ratio (95% CI) | P-value |
| No Methylprednisolone | 39.4% (13/33) | - | - |
| Methylprednisolone with or without any other treatments | 31.3% (5/16) | 0.64 (0.13 – 2.80) | 0.56 |
| No IVIG | 27.3% (9/33) | - | - |
| IVIG with or without any other treatments | 56.3% (9/16) | 1.88 (0.46 – 7.92) | 0.38 |
| Without combination of Methylprednisolone and IVIG | 40.5% (17/42) | - | - |
| With combination of Methylprednisolone and IVIG | 14% (1/7) | 0.27 (0.01 – 2.07) | 0.27 |

Three adjusted univariate logistic regression models are performed on patients in PedSep-D. In each model, mortality is the

outcome. Covariates of interest in the first model include a binary variable indicating Methylprednisolone usage. Patients without Methylprednisolone are set as reference group. Covariates of interest in the second model include a binary variable indicating IVIG usage. Patients without IVIG are set as reference group. Covariates of interest in the third model include a binary variable indicating Methylprednisolone + IVIG usage. Patients without combination therapy are set as reference group. All three models are adjusted for sex, age, ethnicity, race, and total PRISM score

**eTable 15. Interaction for methylprednisolone, dexamethasone, and IVIG; and, for ECMO and plasma exchange therapies in PedSep-D**

|  | Interaction^a^ | Lower bound | Upper bound | p-value |
| --- | --- | --- | --- | --- |
| Dexamethasone × IVIG | 1.84×10^-9^ | <1×10^-7^ | 1.59×10^6^ | 0.99 |
| Methylprednisolone × IVIG | 0.03 | 5.80×10^-4^ | 0.66 | 0.04 |
| Union of Dexamethasone and Methylprednisolone × IVIG | 0.02 | 3.68×10^-4^ | 0.51 | 0.03 |
| ECMO × Plasma Exchange | 0.02 | 1.65×10^-4^ | 0.97 | 0.06 |

Target therapies are selected based on an unadjusted mortality odds ration < 0.1 in Elastic net analysis in PedSep-D phenotype. All logistic regression models are adjusted for sex, age, ethnicity, race, and total PRISM score.

a. Each interaction is the log of coefficient estimated from a logistic multivariate model. For example, to estimate interaction between Methylprednisolone and IVIG, the covariates in the model involve Methylprednisolone, IVIG, and interaction term Methylprednisolone × IVIG. Significant results for Methylprednisolone × IVIG term can be interpreted as the association of IVIG with mortality was modified by exposure to Methylprednisolone in the PedSep-D patients.

**eTable 16. Three univariable adjusted logistic regression models comparing the effects of Methylprednisolone alone, IVIG alone, and Methylprednisolone + IVIG together to reference group (none of these treatments) in PedSep-B,C,D**

|  | PedSep-B | | | PedSep-C | | | PedSep-D | | |
| --- | --- | --- | --- | --- | --- | --- | --- | --- | --- |
|  | Mortality rate (N) | Odds ratio (95% CI) | P-value | Mortality rate (N) | Odds ratio (95% CI) | P-value | Mortality rate (N) | Odds ratio (95% CI) | P-value |
| No Methylprednisolone or IVIG (reference group) | 12% (5/49) | - | - | 0% (0/41) | - | - | 29% (7/24) | - | - |
| Methylprednisolone alone | 5% (1/19) | 0.19 (0.01-1.06) | 0.10 | 33% (5/15) | NA | NA | 44% (4/9) | 1.27 (0.31-5.16) | 0.73 |
| IVIG alone | 33% (2/6) | 6.00 (1.00-36.78) | 0.04 | 30% (3/10) | NA | NA | 67% (6/9) | 4.09 (1.07-2.03) | 0.053 |
| Methylprednisolone + IVIG | 25% (1/4) | 1.62 (0.12-11.14) | 0.65 | 33% (3/9) | NA | NA | 14% (1/7) | 0.31 (0.003-1.48) | 0.19 |

Three adjusted logistic regression model are performed on patients in PedSep-B,C,D, separately. In each model, mortality is the outcome. Covariates of interest include a categorical variable indicating treatment usage. Four categories of the covariate are patients with 1) Neither Methylprednisolone nor IVIG, 2) Methylprednisolone alone, 3) IVIG alone, 4) both of Methylprednisolone and IVIG. Patients with neither Methylprednisolone nor IVIG are set as reference group. All three models are adjusted for sex, age, ethnicity, race, and total PRISM score. NA – not applicable because 0% mortality in comparator group.

**eTable 17. Interaction between combined treatment (Methylprednisolone + IVIG) and PedSep-D membership**

|  | Interaction^a^ | 95% CI | P-value |
| --- | --- | --- | --- |
| PedSep-D × Methylpred + IVIG | 0.04 | (0.001, 0.56) | 0.026 |

a. Interaction is the log of coefficient of interaction term estimated from a logistic multivariate model. The adjusted logistic regression model is performed on patients in PedSep-B,C,D. Mortality is the outcome of the multivariate model. Covariates of interest include treatment combination (coding 0/1 for using neither Methylprednisolone nor IVIG/using both Methylprednisolone and IVIG), PedSep-D (coding 0/1 for PedSep-B,C/PedSep-D), and their interaction term. The model is adjusted for sex, age, ethnicity, race, and total PRISM score. Significant results for PedSep-D Methylprednisolone × IVIG term can be interpreted as the combination effect of Methylpred and IVIG can be modified by PedSep-D membership of patients.

**eFigure 1. CONSORT diagram of parent study selected for machine learning analysis**


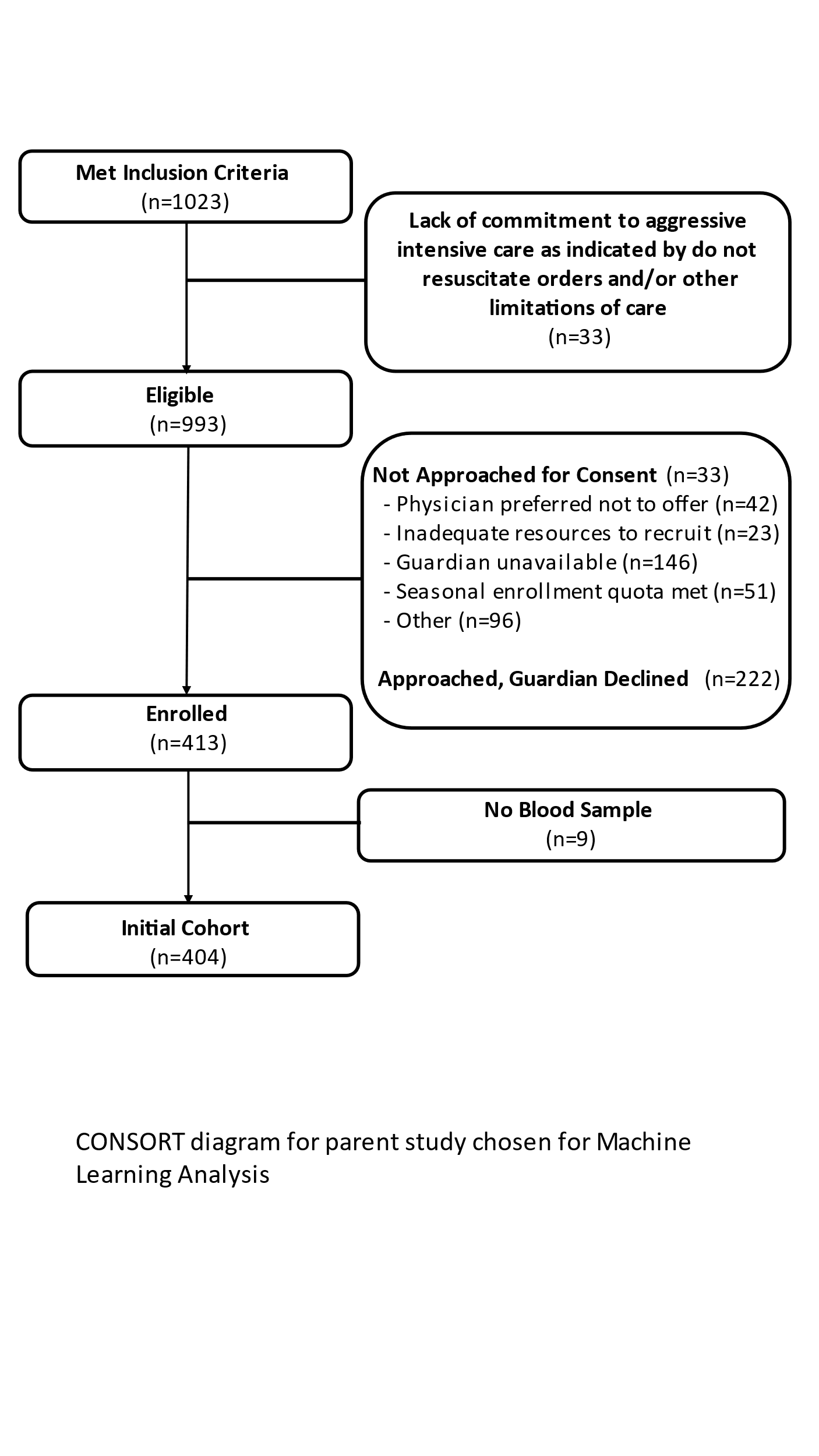


**eFigure 2. Heatmap of correlation between clinical variables for phenotyping**


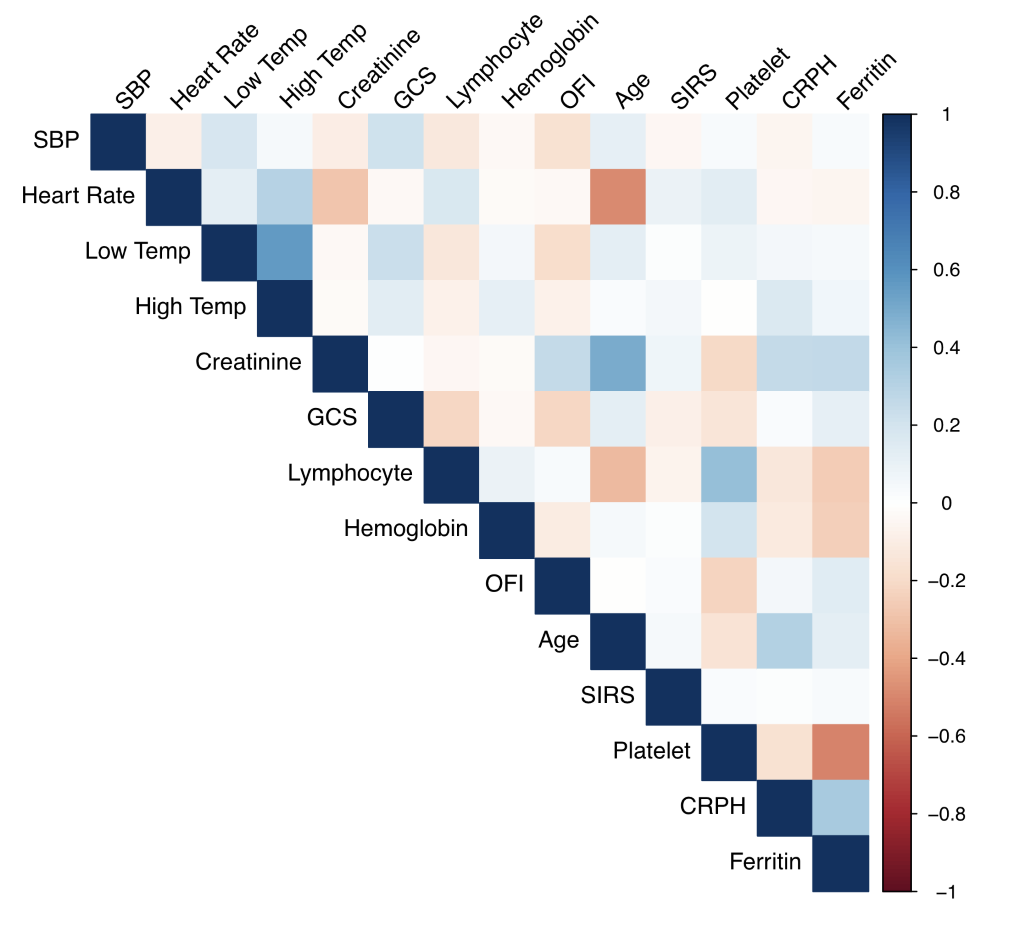


Abbreviations: SBP: Systolic blood pressure; Temp: temperature; GCS: Glasgow coma scale score; OFI, organ failure index; SIRS, systemic inflammatory response syndrome; CRPH, C-reactive protein

Heatmap shows darker color (red or blue) when the Spearman rank order correlation coefficient is greater in positive or negative direction.

**eFigure 3. OPTICS plot (N= 404)**


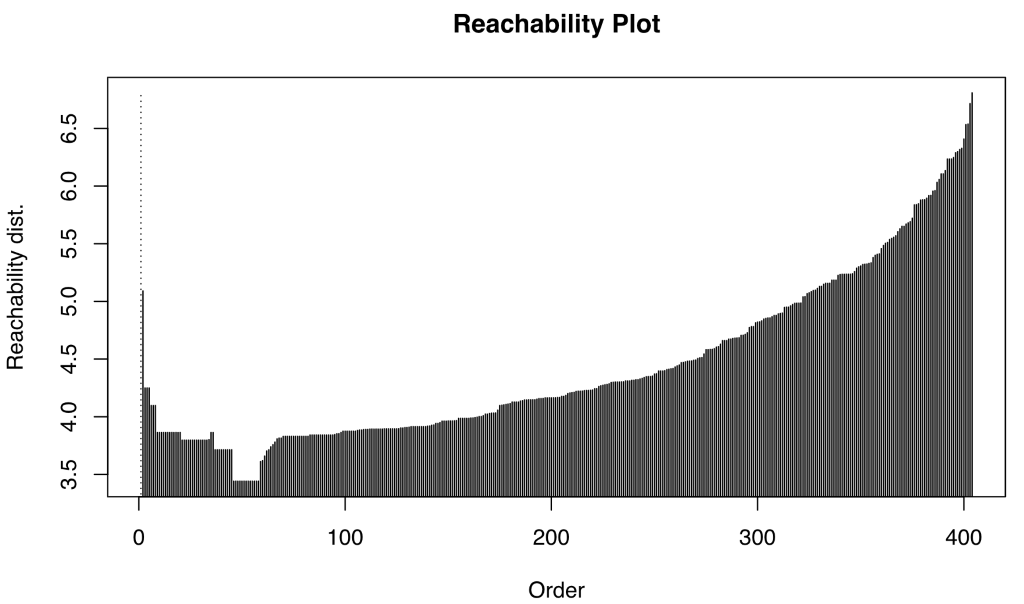


Interpretive example: The OPTICS plot is a figure with the ordering of the patients on the x-axis and the reachability distance on the y-axis. The reachability-distance of two points (samples) is either the distance between them, or the core distance of the core point, whichever is bigger. In our case, the plot shows a smooth rise in reachability distance (as opposed to well demarcated sets). This implies that a partitioning approach such as consensus K means clustering is the preferred statistical algorithm, as opposed to a clustering approach such as hierarchical clustering.

**eFigure 4. Consensus k clustering results**

(A) The consensus matrices have patients as both rows and columns. Each consensus value is the frequency the two patients are assigned to the same phenotype among 1000 iterations. It ranges from 0 (white) to 1 (dark blue). This heat map shows a good partition of patients when k=4, where a clear separation between blue and white chunks is observed. (B) Consensus CDF plot shows the cumulative distribution functions of the consensus matrix for each k (indicated by colors), estimated by a histogram of 100 bins. This figure is used to determine at what number of clusters (i.e. k) the CDF reaches an approximate maximum; thus, consensus and cluster confidence is at a maximum at this k. It is usually used together with the Delta area plot to determine the optimal k. (C) Delta area plot shows the relative change in area under the CDF curve comparing k and k − 1. For k = 2, there is no k -1, so the total area under the curve rather than the relative increase is plotted. This plot allows one to determine the relative increase in consensus and determine k at which there is no appreciable increase. Usually, an “elbow” is one of the indicators of the optimal k. In our case, the elbow happens when k = 4, where increasing k makes little contribution. (D) Tracking plot shows the cluster assignment of patients (columns) for each k (rows) by color. The colors correspond to the colors of the consensus matrix class assignments. Each column indicates patient. This plot indicates patient cluster membership change if one were to use k=2, k=3, k=4, k=5, or k=6. Clusters with an abundance of unstable members (changing colors within a column) suggest an unstable cluster. (E) Cluster-consensus plot shows the cluster-consensus value of clusters at k=2, k=3, k=4, k=5, or k = 6. This is the mean of all pairwise consensus values between a cluster’s members. Cluster is indicated by color following the same color scheme as the cluster matrices and tracking plots. The bars are grouped by k which is marked on the horizontal axis. High values indicate a cluster has high stability and low values indicate a cluster has low stability. We used 0.5 as a cut off for diagnostic purposes, where consensus values of all four clusters are higher than this threshold.

**eFigure 5. Comparison of Variables between consensus k means clustering and LCA**


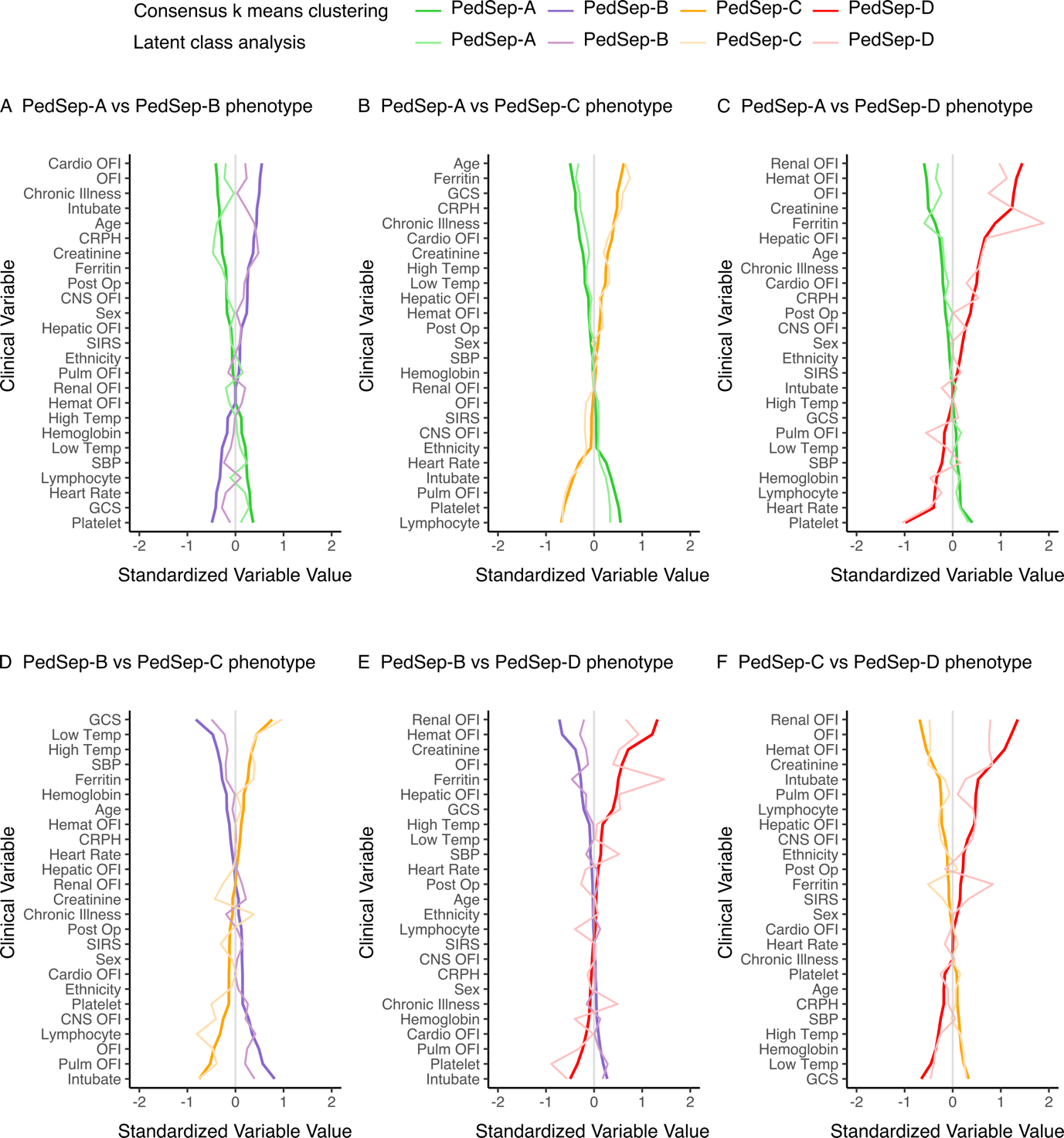


Comparisons of variables that contribute to 24-hour clinical phenotypes using consensus k means clustering and latent class analysis (LCA). In all panels, the variables are standardized such that all means are scaled to 0 and SDs to 1. A value of 1 for the standardized variable value (x-axis) signifies that the mean value for the phenotype was 1 SD higher than the mean value for both phenotypes shown in the graph as a whole. CNS - central nervous system; CRP - C-reactive protein; GCS - Glasgow Coma Scale; Hemat - Hematologic; Intubate- Intubation with endotracheal tube; OFI- organ failure index; Post Op - post-surgery; Pulm- pulmonary; Temp- temperature; SBP- systolic blood pressure; Chronic illness – not previously healthy; Ethnicity – higher number with more non-Hispanic; Sex – higher with more males in group.

**eFigure 6. Sensitivity analysis using latent class clustering (N=404), showing probabilities of PedSep-A, B, C, and D phenotype assignment.**


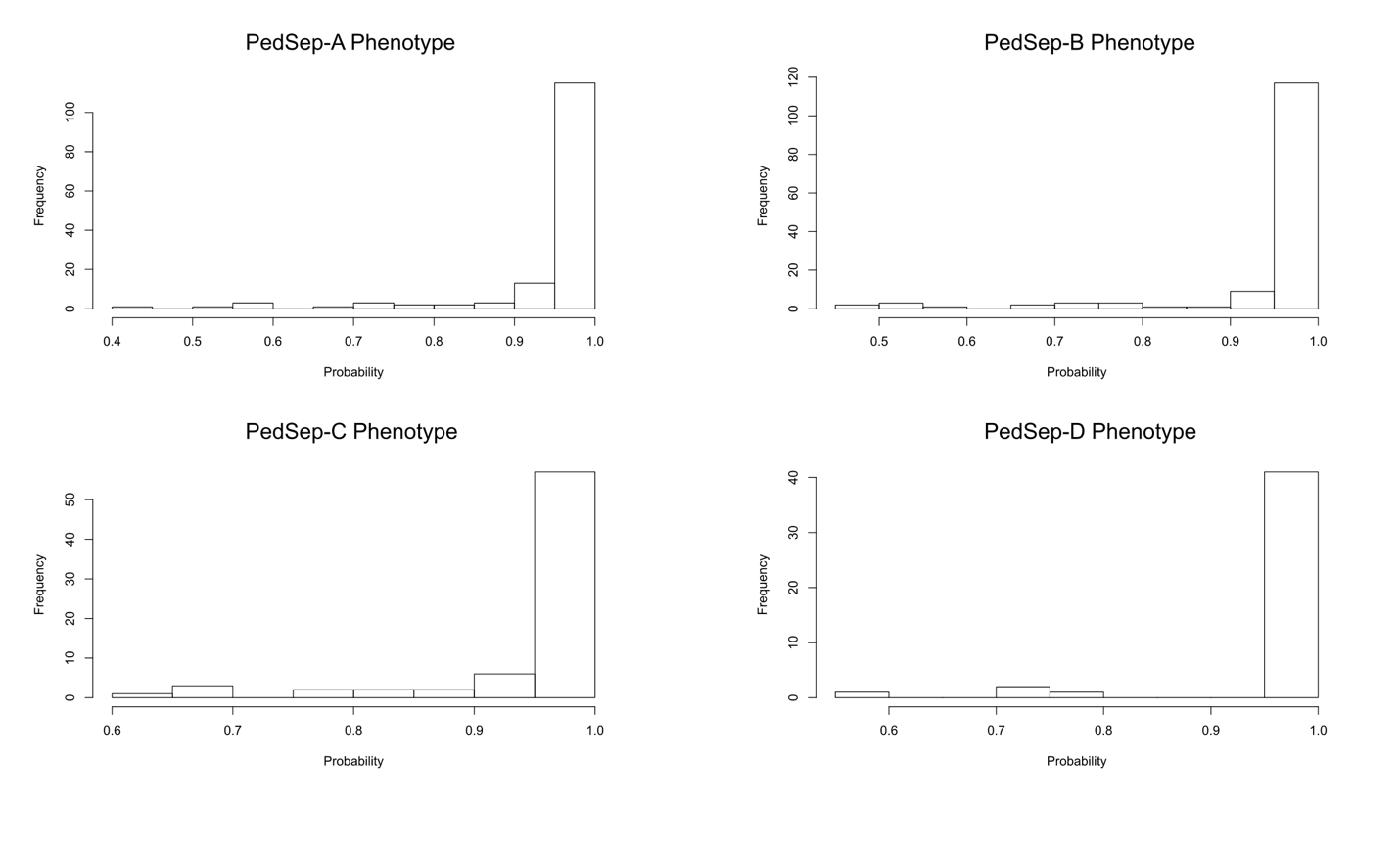


*Interpretive example*: Using latent class analysis to derive phenotypes (called clusters in this output), histograms of within phenotype probability demonstrated that members have high probability of being a phenotype member (>0.9).

**eFigure 7. Comparison of phenotype membership between Consensus k-means and LCA**


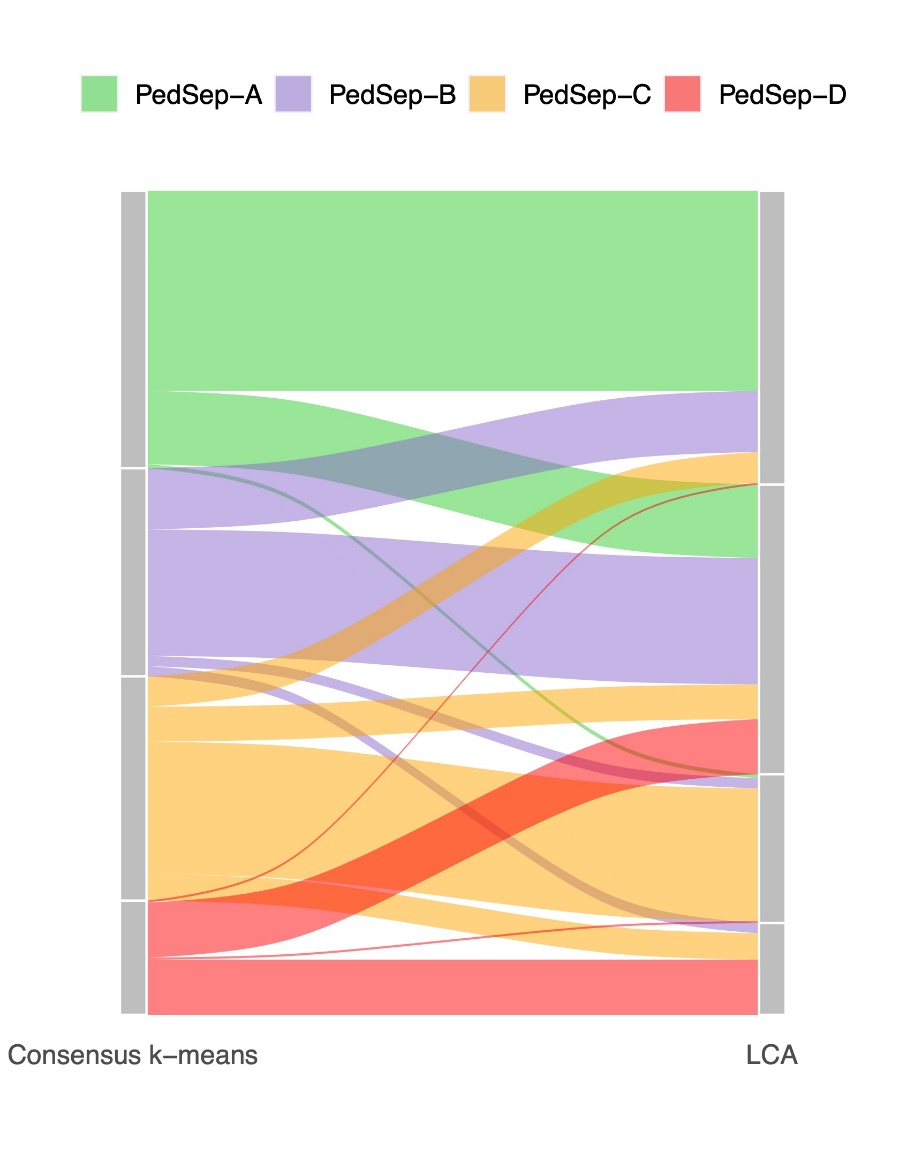


This alluvial diagram shows the phenotype membership difference of 404 patients between Consensus k-means and LCA. The blocks on the two sides represent four phenotypes identified by Consensus k-means and LCA, separately. The streams are colored by phenotypes defined by Consensus k-means. The stream fields between two blocks represent changes in the patient phenotypes assignment in two methods. The height of a block represents the size of the phenotype and the height of a stream field represents the size of the patients contained in both phenotypes connected by the stream field. We observed a consistency of phenotype membership between two cluster methods. While a subset of patients is reassigned in the confirmatory LCA method, the majority of assignment remain robust to method of clustering.

**eFigure 8. t-SNE plot of previously healthy, Leukemia, and cardiovascular + postoperative diagnoses of patients according to phenotypes.**


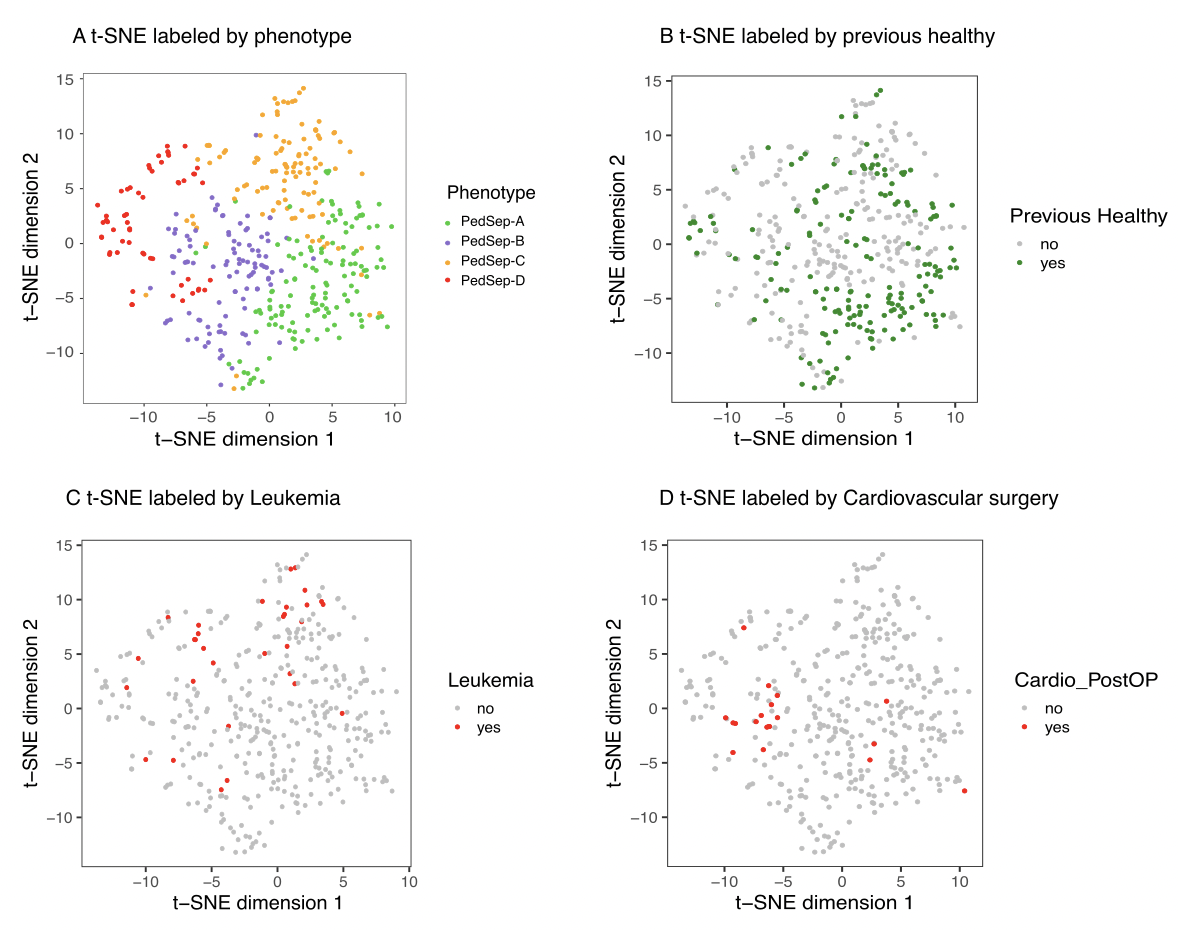


(A) Visualization of phenotypes using t-distributed stochastic neighbor embedding (t-SNE) technique with phenotype shown in color, (B) Previous healthy shown in color, (C) Leukemia shown in color, and (D) Cardiovascular surgery shown in color

**eFigure 9. t-SNE plot of infection status across PedSep-A, B, C, and D**

(A) Visualization of phenotypes using t-distributed stochastic neighbor embedding (t-SNE) technique with phenotype shown in color, (B) Bacterial infection shown in color, (C) Viral infection shown in color, and (D) Fungal infection shown in color

**eFigure 10. Inflammatory Cytokines Across PedSep A, B, C, and D**


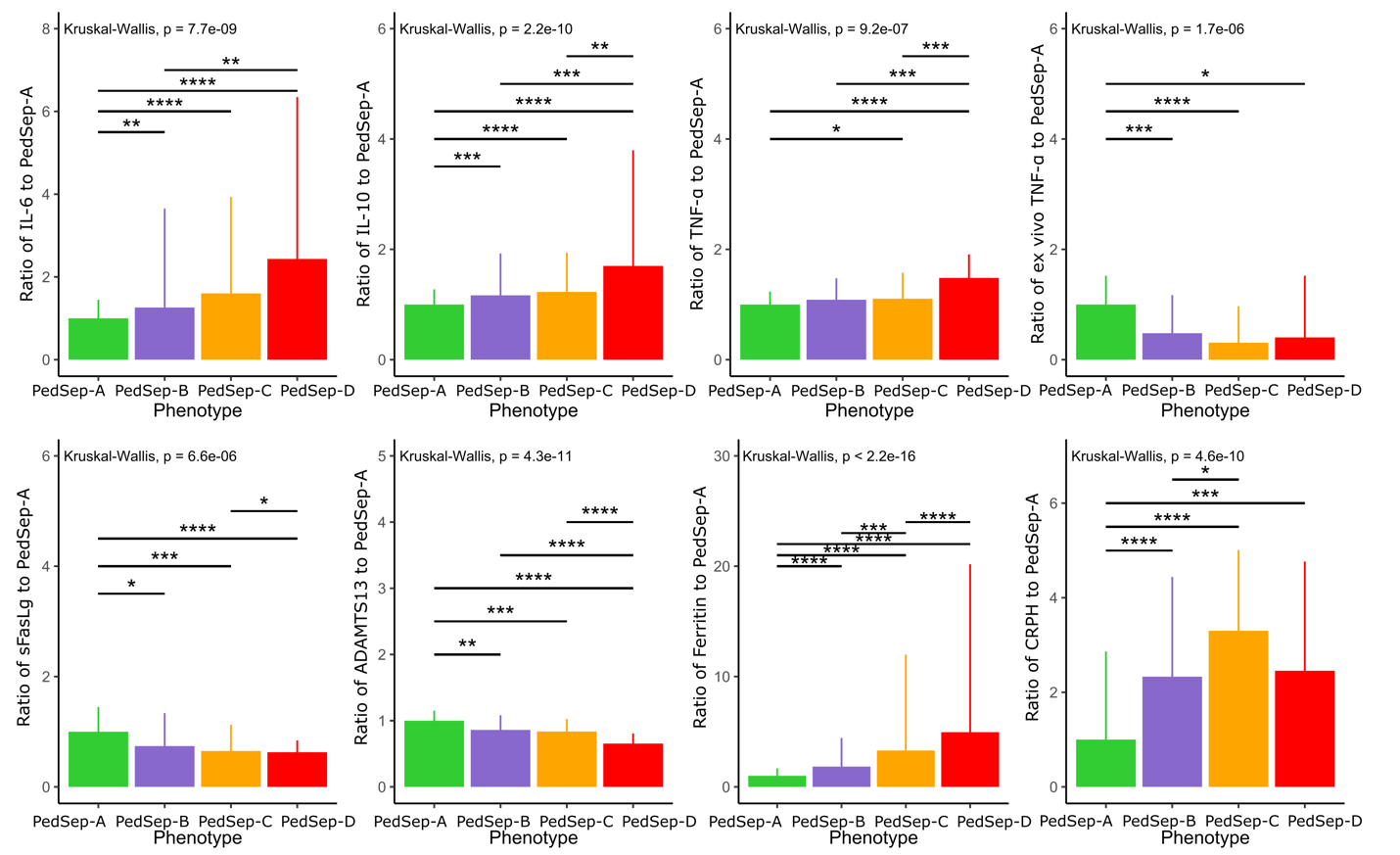


Ratio of each biomarker was calculated as the cytokine value standardized by the median value for the Ped-α phenotype (reference group). All comparisons within data sets across phenotypes were significant (*P*< .001). Errors bars indicate the upper bound of the interquartile range of the biomarker standardized by the median value for the PedSep-A phenotype. Inflammatory cytokines IL-6, IL-10, and TNF measured at baseline were greater in the Ped-C phenotype (orange) and Ped-D phenotype (red) compared with the Ped-A phenotype (green), suggesting a predominantly hyperinflammatory response. TNF indicates tumor necrosis factor.

**eFigure 11. Alluvial plot showing distribution of PedSep-A,B,C,D and across baseline OFI (N=404)**


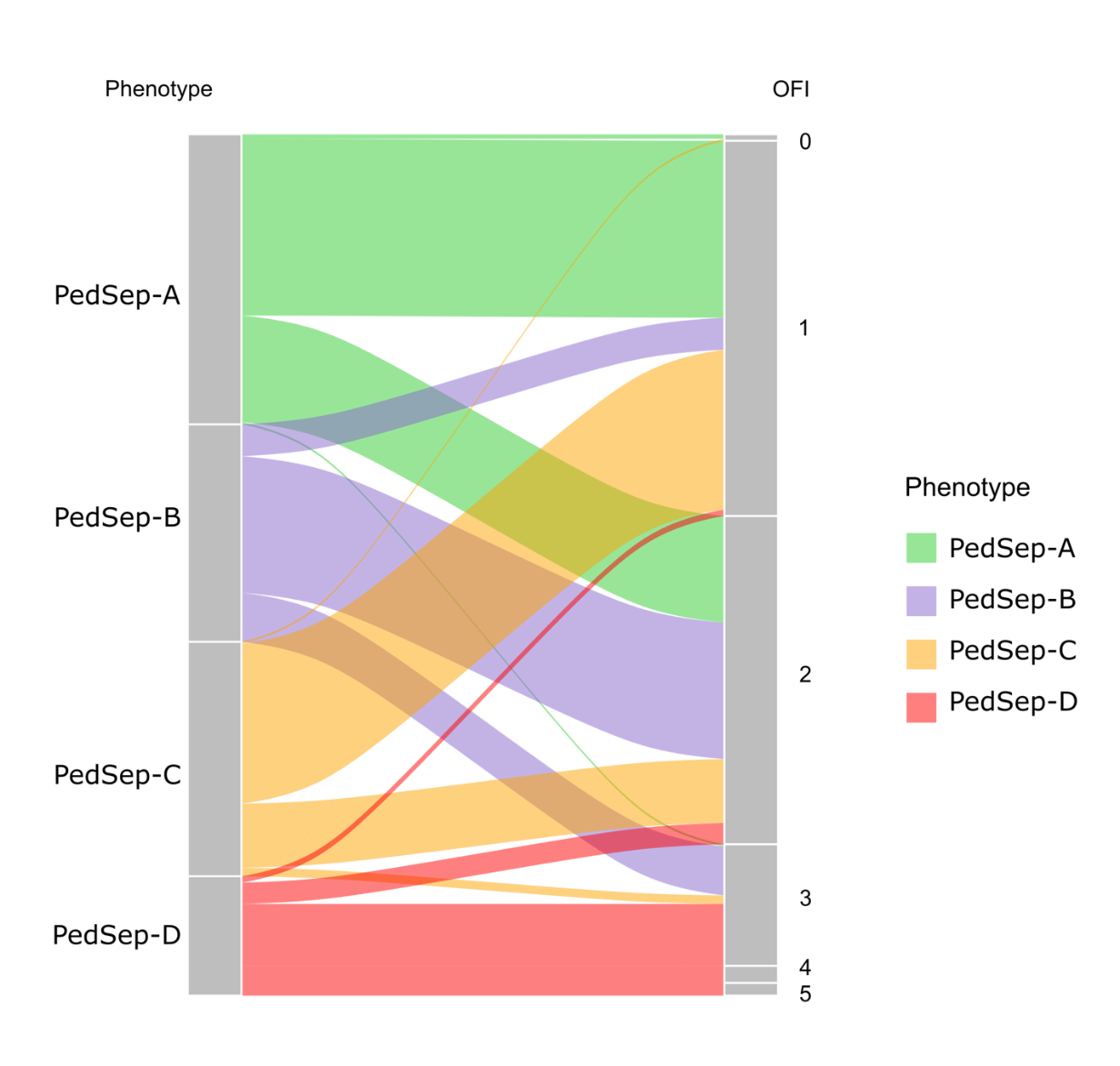


In these alluvial plots, phenotype members are shown by color on the left column and distribute across OFI in the right column. In general, most of PedSep-A and PedSep-C phenotype distributed across lower OFI (OFI less or equal to 2), while phenotype PedSep-B and PedSep-D distributed across higher OFI (OFI is higher or equal to 2 for PedSep-B, OFI is higher or equal to 3 for PedSep-D).

**eFigure 12. Comparison of Day 1 Biomarkers That Contribute to Outcome in PedSep-A, B, C, and D**

In all panels, the variables are standardized such that all means are scaled to 0 and SDs to 1. A value of 1 for the standardized variable value (x-axis) signifies that the mean value for the phenotype was 1 SD higher than the mean value for both phenotypes shown in the graph as a whole.

**eFigure 13.** **Organ failure curve over 28 days among phenotypes assigning 0 for survivors and 6 for non-survivors at discharge**


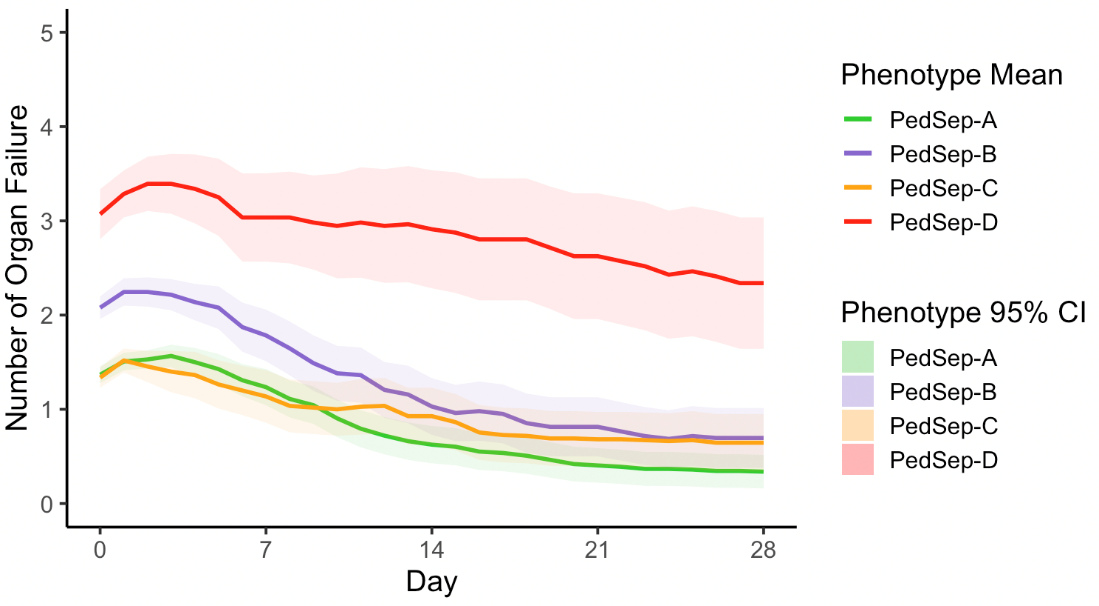


The figure is generated with a nesting strategy where the OFI for those who died and left the PICU is assigned at 6 organ failures for the later time points; and for those who are alive when leaving the PICU is assigned at 0 organ failures for the later time points.

**eFigure 14. t-SNE plot of outcomes (N = 404) across PedSep-A, B, C, and D**

 

Abbreviations:  TAMOF, thrombocytopenia associated multiple organ failure; IPMOF, Immunoparalysis associated multiple organ failure; SMOF, sequential liver failure associated multiple organ failure; MAS, macrophage activation syndrome

(A) Visualization of phenotypes using t-distributed stochastic neighbor embedding (t-SNE) technique with phenotype shown in color, (B) mortality shown in color, (C) TAMOF shown in color, (D) IPMOF shown in color, (E) SMOF shown in color, and (F) MAS shown in color

**eFigure 15. Counts of patients receiving 14 therapies alone and in combination**

This heatmap shows the count of treated patients (A) and survivors among treated patients (B) in PedSep-B, C, and D. Therapies not associated with outcomes in univariable analysis (eTable 12) were not included. Drugs and Organ Support treatments are sorted in alphabetical order.
